# Effect of atorvastatin on the gut microbiota of high fat diet-induced hypercholesterolemic rats

**DOI:** 10.1038/s41598-017-19013-2

**Published:** 2018-01-12

**Authors:** Tariq Jamal Khan, Youssri M. Ahmed, Mazin A. Zamzami, Saleh A. Mohamed, Imran Khan, Othman A. S. Baothman, Mohamed G. Mehanna, Muhammad Yasir

**Affiliations:** 1 Biochemistry Department, Faculty of Science, King Abdulaziz University, Jeddah, 21452 Saudi Arabia; 2 State Key Laboratory of Quality Research in Chinese Medicine, Macau University of Science and Technology, Macau, 999078 China; 3 Special Infectious Agents Unit, King Fahd Medical Research Center, King Abdulaziz University, Jeddah, 21452 Saudi Arabia

## Abstract

The aim of the present study was to investigate alterations in gut microbiota associated with hypercholesterolemia and treatment with atorvastatin, a commonly prescribed cholesterol-lowering drug. In this study, seven experimental groups of rats were developed based on diets [high-fat diet (HFD) and normal chow diet (NCD)] and various doses of atorvastatin in HFD and NCD groups. 16S rRNA amplicon sequencing was used to analyze the gut microbiota. Atorvastatin significantly reduced the cholesterol level in treated rats. Bacterial diversity was decreased in the drug-treated NCD group compared to the NCD control, but atorvastatin-treated HFD groups showed a relative increase in biodiversity compared to HFD control group. Atorvastatin promoted the relative abundance of Proteobacteria and reduced the abundance of Firmicutes in drug-treated HFD groups. Among the dominant taxa in the drug-treated HFD groups, *Oscillospira*, *Parabacteroides*, *Ruminococcus*, unclassified CF231, YRC22 (Paraprevotellaceae), and SMB53 (Clostridiaceae) showed reversion in population distribution toward NCD group relative to HFD group. Drug-treated HFD and NCD groups both showed an increased relative abundance of *Helicobacter*. Overall, bacterial community composition was altered, and diversity of gut microbiota increased with atorvastatin treatment in HFD group. Reversion in relative abundance of specific dominant taxa was observed with drug treatment to HFD rats.

## Introduction

Metabolic syndrome has become a global epidemic, and among the associated disorders, hypercholesterolemia is a particular focus of attention because of its cost burden on health care systems worldwide^1^. According to the World Health Organization, an estimated 26 million deaths occur annually because of hypercholesterolemia-related cardiovascular diseases (CVDs)^2^. A high-fat diet (HFD) has been shown to alter the species composition of intestinal bacteria (i.e., gut microbiota, GM), which in turn play a crucial role in the development of obesity, insulin resistance, and other disorders associated with metabolic syndrome^3,4^. For example, an increase in bacteria belonging to the phylum Firmicutes and a reduction in those belonging to Bacteroidetes were observed in obese individuals and in an animal model^4,5^. Firmicutes bacteria are rich in enzymes responsible for the synthesis of short-chain fatty acids (SCFAs)^6^. GM-produced SCFAs are associated with increased nutrient uptake and energy harvest via G-protein-coupled receptor activation. Hence, they support the development of obesity^6^. Association studies have suggested that GM could affect cholesterol metabolism and also interact with cholesterol-lowering drug like pravastatin and simvastatin^7^. The significance of GM with regard to the metabolism and retention of cholesterol was indicated in a study of germ-free mice in which the absence of GM was associated with enhanced cholesterol excretion in stool^8^. *Lactobacillus reuteri* (NCIMB 30242), a potentially probiotic bacterium, lowered the low-density lipoprotein cholesterol (LDL-C) level and increased the total bile acid level in hypercholesterolemic patients, highlighting the importance of bacteria in regulating the level of cholesterol^9^. Bile acids (for which cholesterol is the precursor molecule) can alter the GM composition, and intestinal bacteria were also reportedly involved in bile acid metabolism, suggesting that GM may be altered by changes in the cholesterol level^10^. Consequently, it is important to understand the alteration in GM in response to hypercholesterolemia and the presence of cholesterol-lowering drugs.

Atorvastatin is a commonly used cholesterol-lowering drug to treat hypercholesterolemia. It inhibits 3-hydroxy-3-methylglutaryl-coenzyme A reductase (HMG-CoA), the enzyme that catalyzes the rate-limiting step of cholesterol synthesis^11^. To the best of our knowledge, no published study has linked GM alteration with atorvastatin treatment in a HFD-induced hypercholesterolemic rat model. We aimed to investigate how the GM responds to an atorvastatin-induced reduction in cholesterol level in a HFD-induced hypercholesterolemic rat model. In particular, we sought to discover whether the drug treatment could affect the GM composition and diversity in hypercholesterolemic rats relative to healthy controls.

## Results

### Hypercholesterolemia and drug treatment analysis

**Figure 1:**
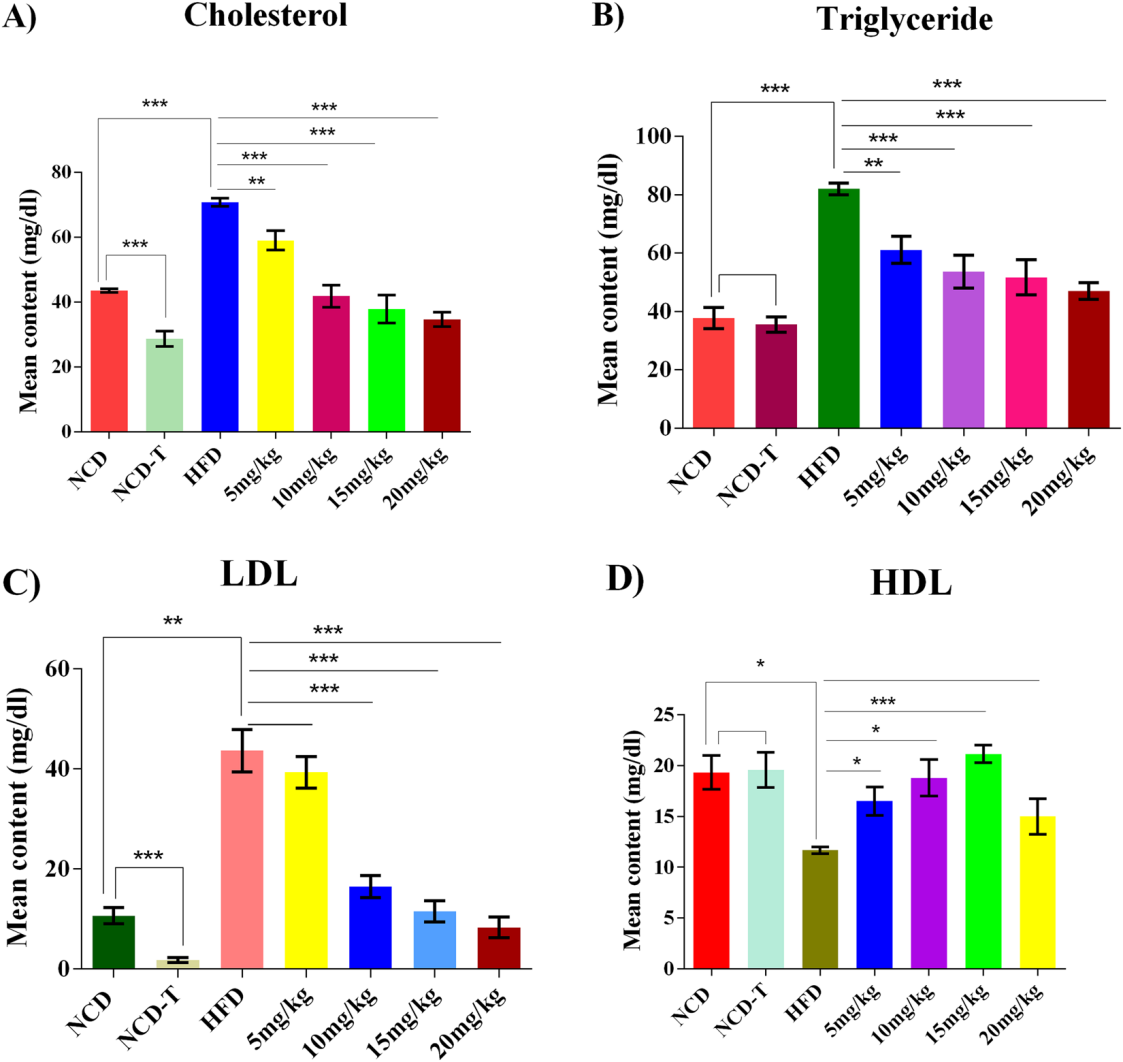
Comparison of serum lipid profiles among experimental groups of rats. (**A**) Cholesterol level, (**B**) triglyceride level, (**C**) low-density lipoprotein (LDL) level, and (**D**) high-density lipoprotein (HDL) level. The x-axis shows the groups, and the y-axis displays the level of mean content in mg/dl. The NCD was the reference for NCD-T and HFD, while HFD was the reference for 5 mg/kg, 10 mg/kg, 15 mg/kg, and 20 mg/kg. NCD, normal chow diet; NCD-T, normal chow diet + atorvastatin treatment; HFD, high-fat diet; 5 mg/kg, HFD + 5 mg/kg atorvastatin; 10 mg/kg, HFD + 10 mg/kg atorvastatin; 15 mg/kg, HFD + 15 mg/kg atorvastatin; 20 mg/kg, HFD + 20 mg/kg atorvastatin. *P < 0.05; **P < 0.01 and ***P < 0.001, represent the significance level between the groups.

Hypercholesterolemia was confirmed at 5 weeks of HFD feeding by elevated serum cholesterol levels. Cholesterol (*P* < 0.0001), triglyceride (TG; *P* < 0.0001), and LDL (*P* < 0.05) levels were significantly higher in the HFD group relative to the NCD control group (Fig. 1A–C). In addition, high-density lipoprotein (HDL) levels were significantly lower (*P* < 0.05) in HFD-fed rats compared to NCD controls (Fig. 1D). Atorvastatin treatment for 28 days with various doses in HFD groups significantly lowered the serum levels of cholesterol and TG in the 10 mg/kg, 15 mg/kg, and 20 mg/kg groups (*P* < 0.0001) and in the 5 mg/kg group (*P* < 0.001) compared to the HFD control (Fig. 1A and B). The LDL level was found to be significantly (*P* < 0.0001) decreased in the 10 mg/kg, 15 mg/kg, and 20 mg/kg groups, relative to the HFD group, except in the 5 mg/kg group, for which the difference was insignificant (Fig. 1C). Moreover, significant decrease (*P* < 0.0001) in cholesterol and LDL levels were found in the drug-treated NCD (NCD-T) group relative to the NCD control group (Fig. 1A and C). However, no significant changes were observed in the TG and HDL levels between the NCD and NCD-T groups (Fig. 1B and D).

### Core microbiota

**Figure 2:**
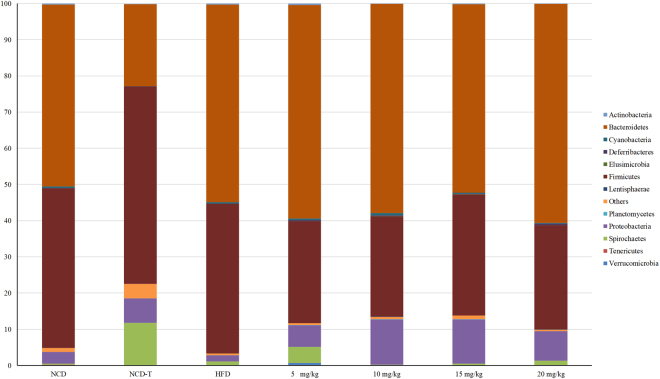
Relative abundance expressed as a percentage of the dominant and/or commonly present bacterial phyla among different diet and drug-treated groups. The cutoff points for selecting the dominant phyla was set to ≥1%, and commonly present taxa were detected in >50% of rats per group. NCD, normal chow diet; NCD-T, normal chow diet + atorvastatin treatment; HFD, high-fat diet; 5 mg/kg, HFD + 5 mg/kg atorvastatin; 10 mg/kg, HFD + 10 mg/kg atorvastatin; 15 mg/kg, HFD + 15 mg/kg atorvastatin; 20 mg/kg, HFD + 20 mg/kg atorvastatin.

Around 1.8 million high-quality reads (>250 bp length) were obtained by using the MiSeq system from the metagenomic DNA extracted from the cecal contents of the slaughtered rats. The sequences were assigned to 28 different phyla including three candidate phyla and the archaeal phylum Euryarchaeota, which was detected at a relatively minor abundance. Ten phyla were commonly present (taxa present in >50% of rats per group) in all groups, but only three phyla were dominant (≥1% of relative abundance). The distribution of these three phyla varied in relative abundance among the HFD and drug-treated HFD groups (5 mg/kg, 10 mg/kg, 15 mg/kg, 20 mg/kg), and the NCD-T group compared to the NCD control group. The majority of sequencing reads belonged to the phyla Firmicutes, Bacteroidetes, and Proteobacteria, which dominated the GM in the different groups (Fig. 2 and Supplementary Table 1). The ratio of Firmicutes to Bacteroidetes was similar in the HFD and NCD groups, but it decreased in a dose-dependent manner in the drug-treated HFD groups compared to the HFD group and increased in the NCD-T group relative to the NCD control. In total, 220 families were identified from the sequence reads (Supplementary Table 2). Prevotellaceae, Ruminococcaceae, Clostridiaceae, Paraprevotellaceae, Bacteroidaceae, and Lachnospiraceae were the relatively abundant families among all groups (Supplementary Fig. 1). Moreover, 535 operational taxonomic unit (OTUs) were detected at the genus level. Among them, only 367 OTUs were classified to different genera (Supplementary Table 3). All groups shared a core set of bacterial genera that were present at ≥1% of relative abundance at least in one group, including *Prevotella*, *Bacteroides*, *Helicobacter*, *Oscillospira*, and *Ruminococcus* (Supplementary Fig. 2).

### Gut microbiota alteration in response to HFD

We found that Proteobacteria was noticeably decreased in the HFD group compared to the NCD group (Fig. 2). Ten phyla present in >50% rats of the HFD group also occurred in the NCD groups, whereas Acidobacteria, Chlamydiae, Fibrobacteres and Fusobacteria were present only in the NCD group (Supplementary Fig. 3). In total, 56 families were shared by both HFD and NCD groups, where three families were reportedly unique to HFD group, and 76 families were detected only in the NCD group (Supplementary Table 4). Moreover, substantially decreased relative abundance of the families Ruminococcaceae, Paraprevotellaceae, Porphyromonadaceae, Desulfovibrionaceae, and Odoribacteraceae were observed in the HFD group compared to the NCD group (Supplementary Fig. 1). Among dominant families, relative abundances of the Prevotellaceae, Bacteroidaceae, and Lachnospiraceae remained unaltered in the HFD group compared to the NCD control (Supplementary Fig. 1). In the GM of the NCD group, we found 69 genera that were depleted in the HFD group. Nine genera that were specifically detected in the HFD group were not present in the NCD group. A considerable increase was observed in the relative abundance of bacteria from the genera *Lactobacillus*, *Treponema*, *Ruminococcus*, *Coprococcus*, and *Clostridium* in the HFD group compared to the NCD control (Supplementary Fig. 2). Moreover, noticeably decreased relative abundance was observed for *Oscillospira*, *Parabacteroides*, *Paraprevotella*, and *Odoribacter* in the HFD group compared to the NCD group. Among the dominant genera, the relative abundance of the *Bacteroides* and *Turicibacter* remained unchanged in the HFD group compared to the NCD control (Supplementary Table 3).

### Dose-dependent alteration of taxa in the atorvastatin-treated groups versus controls

**Figure 3:**
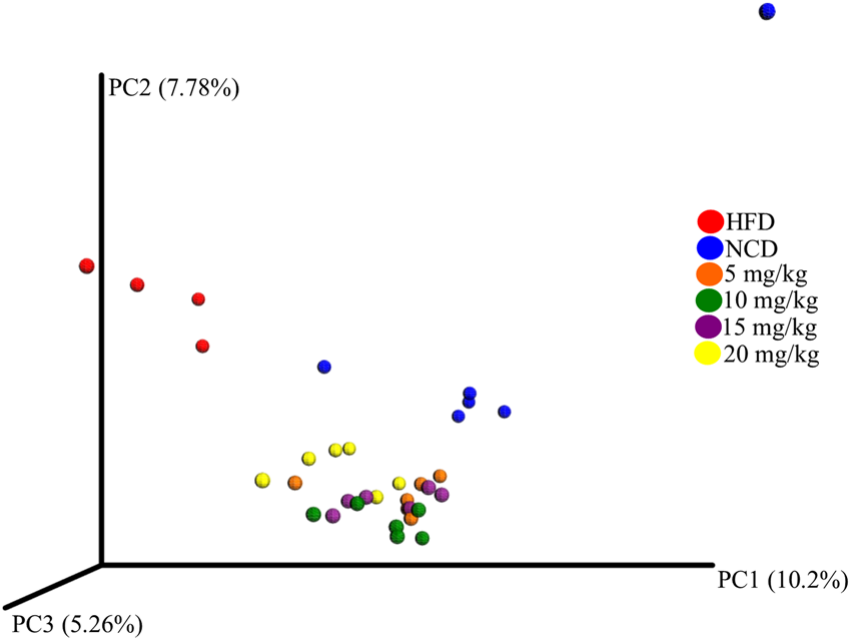
The multivariate principal coordinate analysis of the composition of microbial communities between the different groups. PCoA was performed from the sequences at OTU level with >97% similarity using unweighted UniFrac distance metric. NCD, normal chow diet; NCD-T, normal chow diet + atorvastatin treatment; HFD, high-fat diet; 5 mg/kg, HFD + 5 mg/kg atorvastatin; 10 mg/kg, HFD + 10 mg/kg atorvastatin; 15 mg/kg, HFD + 15 mg/kg atorvastatin; 20 mg/kg, HFD + 20 mg/kg atorvastatin.

**Figure 4:**
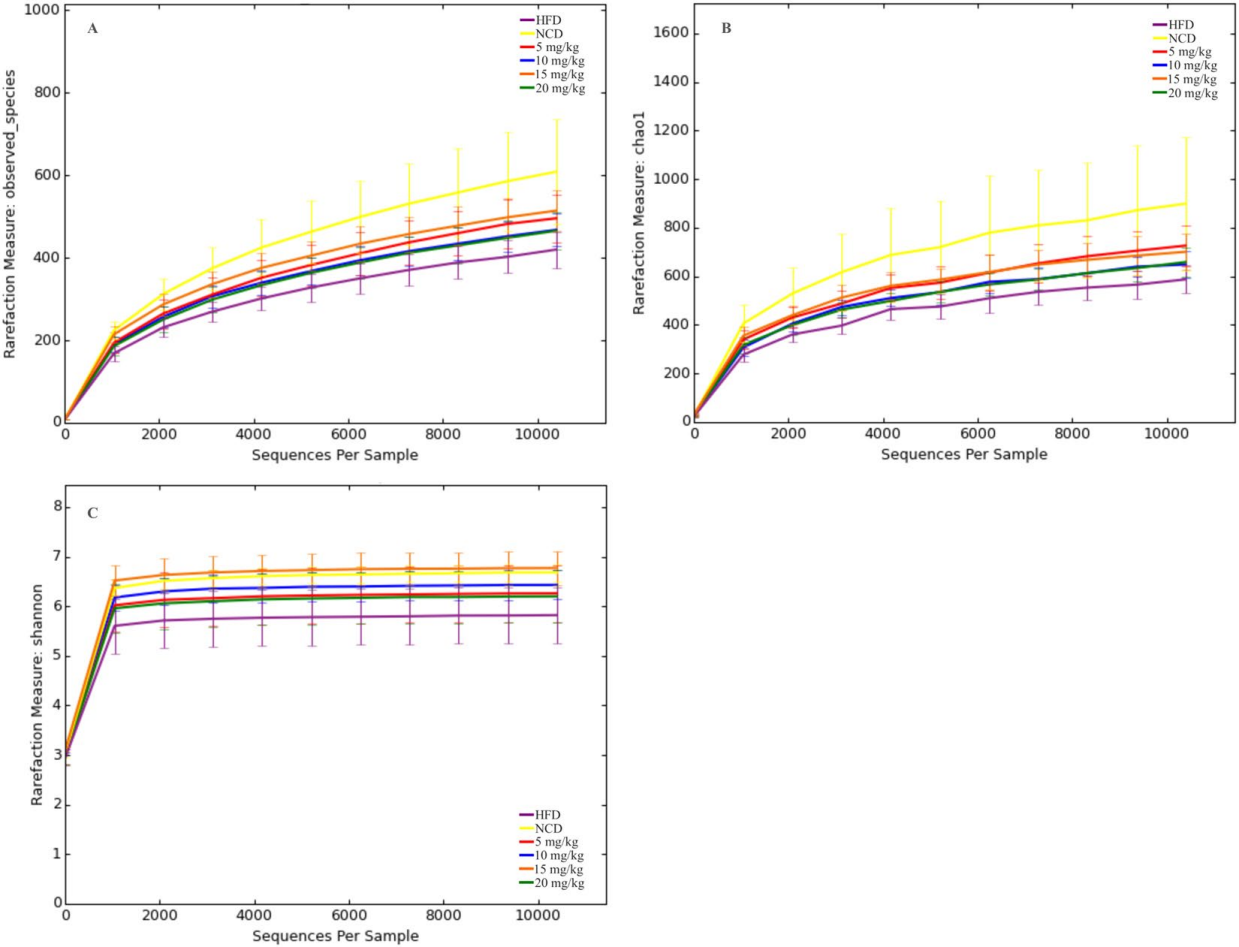
Alpha diversity analysis of the sequence reads. (**A**) Rarefaction curves of the experimentally observed OTUs versus (**B**) estimated by Chao1. (**C**) Biodiversity estimated by Shannon index. NCD, normal chow diet; NCD-T, normal chow diet + atorvastatin treatment; HFD, high-fat diet; 5 mg/kg, HFD + 5 mg/kg atorvastatin; 10 mg/kg, HFD + 10 mg/kg atorvastatin; 15 mg/kg, HFD + 15 mg/kg atorvastatin; 20 mg/kg, HFD + 20 mg/kg atorvastatin.

The relative abundance of Proteobacteria was significantly (*P* < 0.05) increased in the drug-treated HFD groups compared to the HFD group (Fig. 2). However, different doses of the drug decreased the Firmicutes abundance to an almost equal extent compared to the HFD group. The relative abundance of Bacteroidetes was not altered in the drug-treated HFD groups compared to the HFD and NCD groups. In contrast to the HFD group, sequences for Acidobacteria, Fusobacteria, Gemmatimonadetes, Nitrospirae, Chlamydiae, Chlorobi, and Armatimonadetes were retrieved in the drug-treated HFD groups (Supplementary Table 1). These phyla were also present in the NCD group, with the exception of Chlorobi, which was specifically detected in two of the drug-treated HFD groups (5 mg/kg and 10 mg/kg). Analysis of the identified families indicated that the relative abundance of the Ruminococcaceae, Bacteroidaceae, Porphyromonadaceae, Helicobacteraceae, Paraprevotellaceae, Desulfovibrionaceae, and Alcaligenaceae were increased in dose-dependent manner in the drug-treated HFD groups compared to the HFD control (Supplementary Fig. 1). In contrast, a decrease was observed with drug treatment in the relative abundance of the families Clostridiaceae, Lachnospiraceae, Lactobacillaceae, Rikenellaceae, Peptostreptococcaceae, Turicibacteraceae, and Staphylococcaceae (Supplementary Fig. 1). We found 72 genera in the drug-treated HFD groups (5 mg/kg, 10 mg/kg, 15 mg/kg, and 20 mg/kg) that were not present in the HFD group, and 27 of them were detected in the NCD group. Moreover, the genera *Bacteroides*, *Oscillospira*, *Paraprevotella*, *Helicobacter*, and *Parabacteroides* showed a considerable increase in the drug-treated HFD groups compared to the HFD group, while the relative abundance of *Turicibacter*, *Clostridium*, *Ruminococcus*, *Coprococcus*, and unclassified SMB53 and YRC22 were decreased in the drug-treated HFD groups relative to the HFD group (Supplementary Fig. 2). Overall, bacterial diversity was increased in association with the different doses of atorvastatin compared to the HFD group. The atorvastatin reverted the relative abundance of several taxa in the drug-treated HFD groups toward NCD GM compared to the HFD group. Principal coordinate analysis revealed that HFD-fed rats treated with different doses of drug were clustered together, and the GM of those rats were closer to the GM of NCD group, compared to those in the untreated HFD group (Fig. 3). The differences between GM of drug-treated HFD groups and the HFD control were particularly evident in the first two principal coordinate axes of the pairwise beta diversities among samples at 97% OTU. The rarefaction with the observed species level and estimated Chao1 showed that the NCD group followed by drug-treated HFD groups had richer and more diverse GM than the HFD groups (Fig. 4A and B). Moreover, the microbial richness estimated by the Chao1 index and biodiversity assessed by a nonparametric Shannon index for comparison among the groups revealed that the GM of drug-treated HFD groups had substantially higher richness and biodiversity than the HFD group (Fig. 4B and C). Moreover, we found that the microbial diversity of the 15 mg/kg drug-treated HFD group was in the same range as the NCD group (Fig. 4C).

As in the drug-treated HFD groups, the relative abundance of the phylum Proteobacteria, families (Porphyromonadaceae, Desulfovibrionaceae and Helicobacteraceae), and genus *Helicobacter* was increased in the NCD-T group compared to the NCD control group. In addition, *Clostridiaceae*, *Lachnospiraceae*, *Spirochaetaceae*, and *Eubacteriaceae* showed a significant increase in the NCD-T group compared to the NCD control (Supplementary Table 2). Similarly, the genera *Treponema*, *Lachnoclostridium*, *Barnesiella*, *Ruminococcus*, *Eubacterium*, *Desulfovibrio*, and *Roseburia* were also increased in the NCD-T group. While among the dominant genera, relative abundance of *Oscillospira*, *Prevotella*, *Bacteroides*, and *Parabacteroides* were decreased in the NCD-T group compared to the NCD control (Supplementary Table 3).

### Correlations between genera and cholesterol level

**Figure 5:**
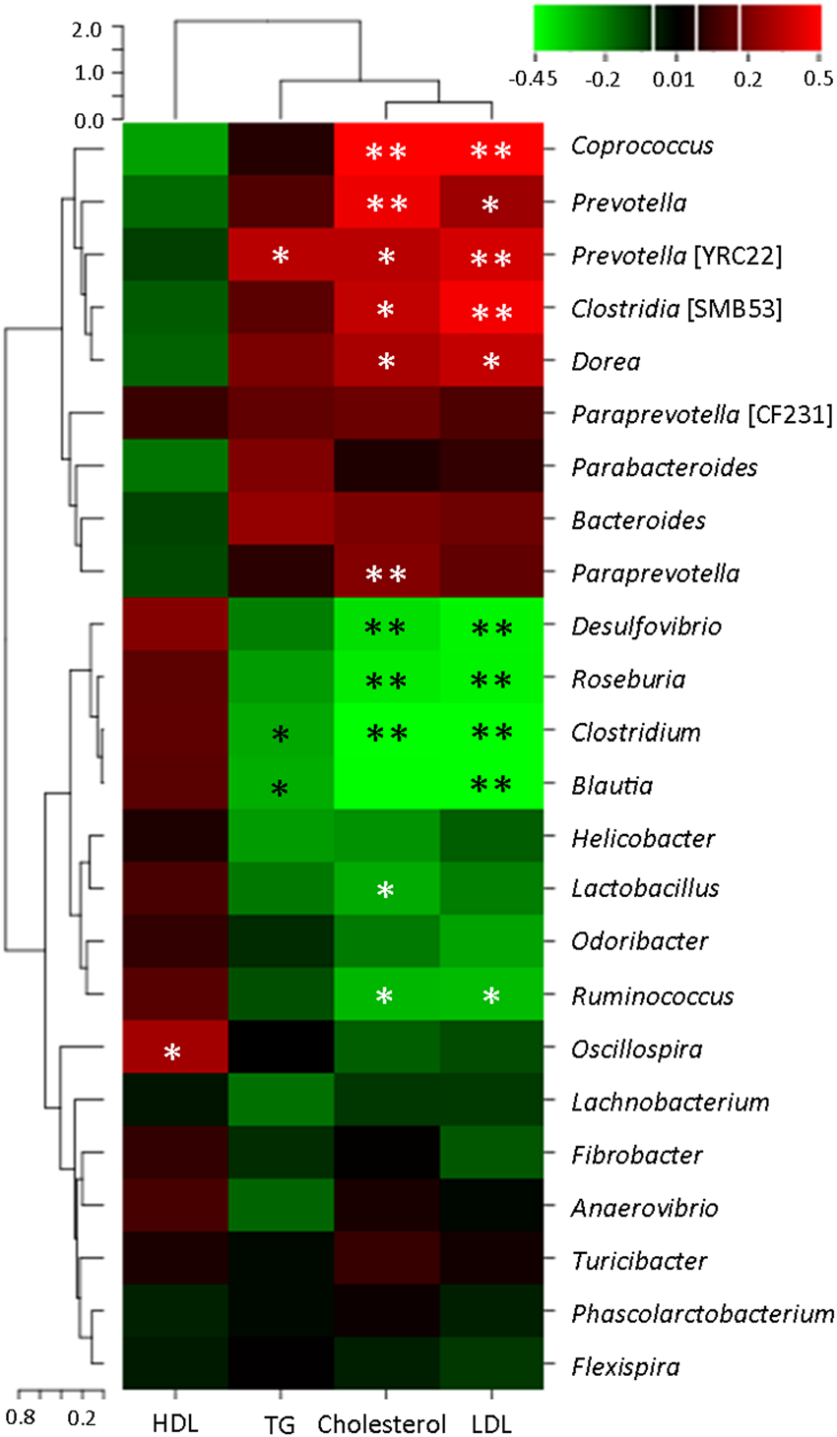
Heat map of the correlation of dominant genera with serum lipid level. The color green indicates a negative correlation, whereas red shows a positive correlation. NCD, normal chow diet; NCD-T, normal chow diet + atorvastatin treatment; HFD, high-fat diet; 5 mg/kg, HFD + 5 mg/kg atorvastatin; 10 mg/kg, HFD + 10 mg/kg atorvastatin; 15 mg/kg, HFD + 15 mg/kg atorvastatin; 20 mg/kg, HFD + 20 mg/kg atorvastatin. *P < 0.05, **P < 0.01 represent the significance level.

Genera correlation with cholesterol level was performed using the Spearman nonparametric correlation analysis. Dominant taxa that were altered in the NCD-T, HFD, 5 mg/kg, 10 mg/kg, 15 mg/kg, and 20 mg/kg groups were found to be correlated with different cholesterols. Genera that were negatively correlated with LDL (ρ > −0.17), TG (ρ > −0.14), and cholesterol (ρ > −0.27) levels includes *Clostridium*, *Desulfovibrio*, *Roseburia*, *Blautia*, *Helicobacter*, *Ruminococcus*, and *Lactobacillus* (Fig. 5). However, *Prevotella*, *Coprococcus*, *Prevotella* [YRC22], *Paraprevotella*, *Clostridia* [SMB53], and *Dorea* were found to be positively correlated with LDL (ρ > 0.2), TG (ρ > 0.1), and cholesterol (ρ > 0.26) levels (Fig. 5). Similarly, *Oscillospira* (ρ > 0.33) showed a positive correlation with the HDL level (Fig. 5).

## Discussion

Recent research showed a convincing link between health abnormalities such as CVD and obesity with GM^12,13^. The GM plays a critical role in the transformation of choline to trimethylamine, which is absorbed by the liver and oxidized by hepatic flavin monooxygenases to produce trimethylamine N-oxide (TMAO), which contributes to CVD development^14,15^. TMAO promotes atherosclerosis, and both TMAO formation and the development of atherosclerosis were suppressed in atherosclerosis-prone mice (C57BL/6 J.Apoe^−/−^) following antibiotic treatment, which highlights the role of GM in CVD pathogenesis^16,17^. Obesity in an HFD-induced obese rat model has been linked with GM dysbiosis^18^. This HFD-mediated dysbiosis was shown to alter parameters such as SCFAs, which may further aggravate the disease pathophysiology^19,20^. Administration of natural extracts including grain sorghum lipid extract and β-glucan (former both possesses cholesterol-lowering ability) and drugs such as berberine, metformin, and simvastatin in animal models caused dysbiosis^18,21–24^. Here, we primarily analyzed the cecum microbial community of HFD and atorvastatin-treated rats and studied the substantial alterations in their bacterial composition. A differential modulation of GM under the influence of the drug was noticed in drug-treated HFD and NCD groups. The bacterial diversity was reduced in the HFD groups but increased with atorvastatin treatment; however, the diversity remained lower in drug-treated HFD rats than in healthy NCD controls. In apo-E^−/−^ mice, a high fat/cholesterol diet lowered the bacterial diversity, and atorvastatin treatment had a considerable effect in modulating the microbiome and metabolome^25^. Similarly, Zhang *et al*. reported reduced bacterial diversity in HFD-fed rats and found that bacterial diversity increased with administration of metformin (drug used to treat type 2 diabetes)^18^, which suggests that metabolic syndrome–associated drugs have a similar effect on bacterial diversity. We found an unaltered ratio of Firmicutes/Bacteroidetes in the HFD group, while the drug-treated HFD groups had a reduced ratio. In a previous HFD-related animal model study, a reduced Firmicutes/Bacteroidetes ratio was reported^26^. However, Zhang *et al*. and Kait Al *et al*. did not find any significant difference in the Firmicutes/Bacteroidetes ratio between HFD and NCD groups^21,27^. The intake of β-glucan–rich wheat flour by hypercholesterolemic patients reduced the Firmicutes/Bacteroidetes ratio^12^. These studies show that the ratio of Firmicutes/Bacteroidetes may not be useful as a marker for metabolic disorder in HFD animal models.

Overall, reduced relative abundance of Clostridiaceae, Lactobacillaceae, Rikenellaceae, Peptostreptococcaceae, and Lachnospiraceae occurred with different doses of atorvastatin treatment in the HFD group. Wang *et al*. reported a reduced level of Clostridiaceae after administration of probiotic bacterial strains (*Lactobacillus paracasei*, *Lactobacillus rhamnosus*, and *Bifidobacterium animalis*) to HFD-fed mice^28^. Similarly, the administration of nonfermentable fiber hydroxypropyl methylcellulose to HFD-fed mice or a switching to a low-fat diet lowered the abundance of Lachnospiraceae^29^. In contrast, an increased abundance of Porphyromonadaceae, Ruminococcaceae, and Desulfovibrionaceae was reported in drug-treated HFD groups. The administration of the *Lactobacillus paracasei* to apo-E^−/−^ mice significantly reduced the cholesterol level and increased the prevalence of Porphyromonadaceae in their feces^30^. The HFD-fed mice supplemented with capsaicin had enhanced abundance of Ruminococcaceae^31^. Similarly, the high-fat/cholesterol diet supplemented with bile salt hydrolase-active *Lactobacillus reuteri* APC 2587 increased the amount of Desulfovibrionaceae^25^.

Atorvastatin treatment in the HFD group increased the relative abundance of the genera *Bacteroides* and *Parabacteroides*. *Bacteroides* and *Parabacteroides* had maximum abundance at a dose of 10 mg/kg and 15 mg/kg, respectively. Similarly, the administration of lingonberries fibers to HFD-fed apoE^−/−^ mice increased the *Bacteroides* and *Parabacteroides* level along with a reduction in the TG level^32^. *Bacteroides* is associated with bile acid metabolism and promotes the deconjugation of taurine-conjugated bile acid present in the serum^18,33^. The high cholesterol/cholic acid diet modified the fecal bile acid composition^34^, and the altered bile acid was reported to be linked with dysbiosis^35^. The level of *Desulfovibrio* (an endotoxin-producing and a sulfate-reducing bacteria)^36,37^ showed an increased relative abundance in the NCD-T group.

Similarly, the genus *Parabacteroides*, which comprises anti-inflammatory bacteria^38^, showed consistent increase in relative abundance at different doses of atorvastatin in the HFD group. *Ruminococcus* (tryptamine-producing bacteria)^39^ showed suppressed level under atorvastatin treatment of HFD-fed rats. The genus *Oscillospira*, which includes butyrate-producing species^40^, showed a consistent increase in drug-treated HFD groups. Treatment with rapamycin (a regulator for energy consumption and storage) in HFD-induced obese rats resulted in an increased abundance of *Turicibacter*^41^. In our study, a decreased level of *Turicibacter* was found in drug-treated HFD groups. Similarly, *Clostridium* (butyrate-producing taxa)^42^ showed reduced level in the drug-treated group. Consistent with our findings, Di Lucci *et. al*. reported an increased abundance of *Coprococcus* in fructose-rich diet–induced metabolic syndrome in an obese rat model^43^.

Catry *et al*. reported an increased level of *Lactobacillus* species after administration of ezetimibe and simvastatin in NCD-fed mice^7^. We observed an increased relative abundance of *Lactobacillus* in the NCD-T group. The cholesterol-lowering ability of bacteria from the genus *Lactobacillus* was previously demonstrated by administration of *Lactobacillus reuteri* LR6, *Lactobacillus reuteri* NCIMB 30242, and *Lactobacillus johnsonii* BS15 in hypercholesterolemic rats and patients and in a mouse model of nonalcoholic fatty liver disease^44–46^. The potential cholesterol-lowering ability of *Lactobacillus* species needs further research to establish its exact role in cholesterol metabolism. We tried to correlate the cholesterol level in rats and the relative abundance of potential cholesterol-lowering bacteria during atorvastatin treatment. A limitation of our study was the lack of metabolomics data. Use of this tool could provide a detailed link between the composition of serum metabolites and the microbiome in the context of atorvastatin treatment.

Overall, this study highlighted the impact of atorvastatin on GM of a control HFD animal model. In particular, atorvastatin administration increased the bacterial diversity and reverted the relative abundance of several dominant taxa that were altered with the HFD toward the NCD phenotype. Atorvastatin treatment also produced a drug-specific effect on the population distribution of certain bacteria in the drug-treated HFD and NCD-T groups. Different classes of cholesterol-lowering drugs may produce varying effects on the gut microbiome and provide a potential link between GM composition, cholesterol level, and drug treatment. In conclusion, cholesterol-lowering drugs that are commonly prescribed during hypercholesterolemia should be analyzed with regard to microbiome-modifying effects that may have important implications for host health.

## Material and Reagents

### Animal model and treatment

Forty-two specific pathogen-free Wistar rats (100–120 g) were provided by the animal house of Biochemistry Department, King Abdulaziz University, Jeddah, Saudi Arabia. The HFD contained 3% cholesterol (Sigma Aldrich Co., USA), 0.2% cholic acid (Sigma Aldrich Co., USA), 15% beef tallow, and 81.8% normal chow. All the added components were taken as the percentage of the total diet. Atorvastatin (Pfizer Inc., USA) was used as a cholesterol-lowering drug^47^. All animal experiments in this study were performed at the animal facilities of Biochemistry Department, Faculty of Science, King Abdulaziz University. The study protocol was approved by the ethical research committee of the Faculty of Medicine at King Abdulaziz University under agreement number (Registration No. HA-02-J-008). The experiment was carried out in accordance with approved guidelines.

The rats were acclimatized to the standardized environmental parameters of the animal house for 7 days, including temperature (21 ± 1 °C), relative humidity (60 ± 10%), and a 12-h day–night cycle. The animal house environmental conditions were maintained till the completion of the experiment. Initially, the 42 rats were randomly divided into two groups and fed either a normal chow diet (NCD; n = 12) or a high-fat diet (HFD; n = 30). Hypercholesterolemia was confirmed after 5 weeks of the HFD treatment. From the NCD group, six rats were treated with 10 mg/kg/d dose of atorvastatin (NCD-T) while the other six rats were kept as NCD controls. In the HFD-induced hypercholesterolemic group, six rats served as HFD controls and the remaining 24 rats were divided into four equal groups and treated with different concentrations of atorvastatin (5, 10, 15, or 20 mg/kg/d). The atorvastatin was suspended in a calculated amount of sterile distilled water using a magnetic stirrer, and the drug was administered intragastrically once every 24 hours using a gavage tube^47^. The animals were treated with atorvastatin for 28 days.

### Blood and tissue sample collection and analysis of cholesterol levels

Blood samples were drawn from the orbital plexus at two time points: at 5 weeks to confirm hypercholesterolemia in the HFD group and before slaughtering to evaluate the effect of drug treatment on cholesterol level in the studied groups. Serum was isolated from the blood samples by centrifugation at 3000 rpm at 4 °C for 15 min. The lipid profile, including serum LDL, cholesterol, TG, and HDL, was assayed by Automatic Biochemical Analyzer (Aeroset 09D0501, American). The cecum contents were collected from all animals immediately after slaughtering and were stored at −80 °C for isolation of DNA.

### 16S rRNA sequencing and data processing

The metagenomic DNA was extracted from the cecal contents using AccuVisBio DNA stool extraction kit (AccuVisBio, Abu Dhabi) according to the manufacturer’s instructions, and the DNA concentration was measured by using the Qubit system (Invitrogen, USA). The samples were sequenced for 16 S rRNA genes, targeting the V3–V4 region with barcoded 341 F and 785 R universal primers following the procedure of Dowd *et al*.^48^. Through a limited PCR cycle, dual-index barcodes and Illumina sequencing adapters were used to join the reads. After purification with Agencourt AMPure beads (Agencourt, USA), libraries were normalized using the Nextera XT protocol. Samples were pooled into a single flow cell for sequencing on the MiSeq sequencing platform (Illumina, San Diego) following the manufacturer’s protocol. Automated cluster generation and paired-end sequencing with dual index reads were performed in a single run with a read length of 2 × 300 bp.

The paired end reads were collected by using PANDAseq, and Raw FASTQ files were obtained from the Illumina MiSeq^49^. Primers and barcodes were removed from the sequences. All reads with ‘N’ and those with sequences <250 bp were deleted, and high-quality sequences were de-replicated^50^. The cleaned sequences were then clustered at k = 10 (97% similarity) followed by deletion of chimeras and singleton reads^51,52^. Finally, OTUs were classified using QIIME 1.9 against a curated database derived from GreenGenes^53^. Sequence data of this study is available in the European Nucleotide Archive under project no. PRJEB23060.

### Statistical analysis

The biodiversity and richness of OTUs were calculated using QIIME 1.9 implemented with nonparametric Chao1 and rarefaction analysis, which showed the uniformity and distribution of OTUs in the different groups. Data was normalized to an equal number of reads per sample, and PCoA was performed from the sequences at OTU level with >97% similarity using unweighted UniFrac distance metric. PCoA plot was visualized by the EMPEROR. One-way ANOVA (for parametric data) and nonparametric Kruskal–Wallis and Mann–Whitney tests (for non-normal data) were performed to identify significantly different bacterial taxa among different groups, while Kolmogorov-Smirnov D test was used to ascertain the normality of data. SPSS version 16 was used for statistical analysis. The differences in cholesterols levels between the control and the treated groups were analyzed by Student’s *t*-test (two-tailed) using GraphPad Prism version 6.01 (Graph Pad Software, San Diego, USA), and the significant differences were indicated as *P < 0.05, **P < 0.01, ***P < 0.001. Correlations between genera and cholesterol level were calculated using the R statistical framework version 3.1.2 with VEGAN package version 2.2–1.

## Electronic supplementary material

Supplementary File
