# Supplementary material for: Effect of atorvastatin on the gut microbiota of high fat diet-induced hypercholesterolemic rats
Source: Sci Rep. 2018 Jan 12;8:662. doi: 10.1038/s41598-017-19013-2 (PMC5766553; doi:10.1038/s41598-017-19013-2)
Supplement: Supplementary file 1 — Supplementary File [file 41598_2017_19013_MOESM1_ESM.doc]

**Effect of atorvastatin on the gut microbiota of high fat diet-induced hypercholesterolemic rats**

Tariq Jamal Khan^1^, Youssri M. Ahmed^1^, Mazin A. Zamzami^1^, Saleh A. Mohamed^1^, Imran Khan^2^, Othman A.S. Baothman^1^, Mohamed G. Mehanna^1^, Muhammad Yasir^3^*

^1^Biochemistry Department, Faculty of Science, King Abdulaziz University, Jeddah, 21452, Saudi Arabia

^2^State Key Laboratory of Quality Research in Chinese Medicine, Macau University of Science and Technology, Macau, 999078, China

^3^Special Infectious Agents Unit, King Fahd Medical Research Center, King Abdulaziz University, Jeddah, 21452, Saudi Arabia

***Corresponding Author: Muhammad Yasir**

E-mail: yasirkhattak.mrl@gmail.com


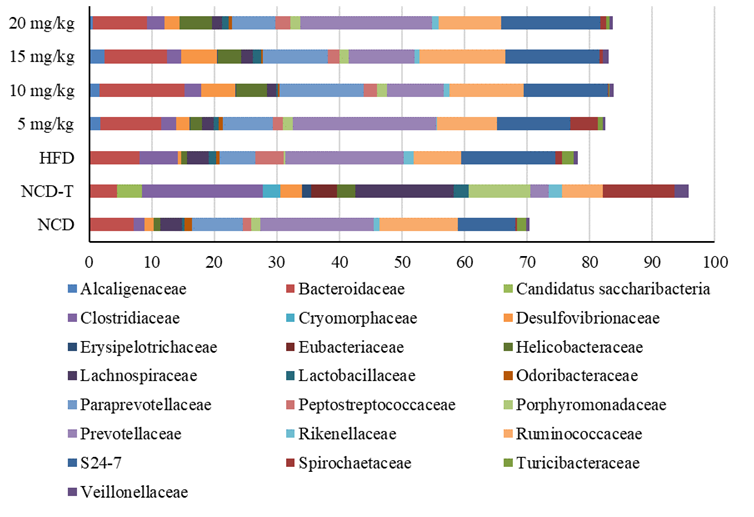


**Supplementary Fig. 1. Relative abundance expressed as a percentage of the families that were dominant and altered with the diet or drug treatment in different groups.**

The cutoff point for comparative analysis among different groups for the dominant families was set to ≥1% detected in at least one group. The *x*-axis shows percentage relative abundance, and the *y*-axis displays the studied groups. NCD, normal chow diet; NCD-T, normal chow diet + atorvastatin treatment; HFD, high-fat diet; 5 mg/kg, HFD + 5mg/kg atorvastatin; 10 mg/kg, HFD + 10 mg/kg atorvastatin; 15 mg/kg, HFD + 15 mg/kg atorvastatin; 20 mg/kg, HFD + 20 mg/kg atorvastatin.


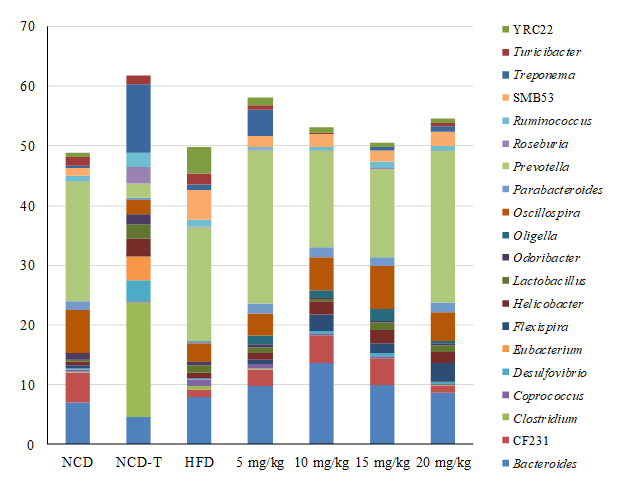


**Supplementary Fig. 2: Comparative analysis of the dominant and altered genera in the gut microbiota of different groups.** The cutoff point for selecting the dominant genera was set to ≥ 1% at least in one group. The *x*-axis shows the groups, and the *y*-axis displays the percentage abundance. NCD, normal chow diet; NCD-T, normal chow diet + atorvastatin treatment; HFD, high-fat diet; 5 mg/kg, HFD + 5mg/kg atorvastatin; 10 mg/kg, HFD + 10 mg/kg atorvastatin; 15 mg/kg, HFD + 15 mg/kg atorvastatin; 20 mg/kg, HFD + 20 mg/kg atorvastatin.


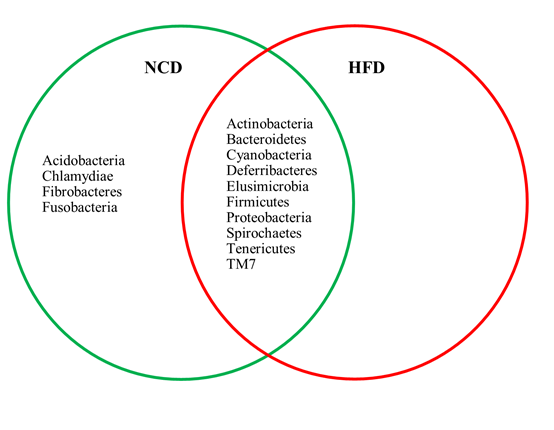


**Supplementary Figure 3: Venn diagram of the shared and unique phyla (present at least in 50% rats of the group) between NCD and HFD groups**. NCD, normal chow diet; HFD, high-fat diet.

**Supplementary Table 1**: Relative abundance expressed in percentage of the identified phyla in this study.

| **Phyla** | **NCD** | **NCD-T** | **HFD** | **5 mg/kg** | **15 mg/kg** | **10 mg/kg** | **20 mg/kg** |
| --- | --- | --- | --- | --- | --- | --- | --- |
| Acidobacteria | 0.285 | 0.000156 | 0 | 0.00084 | 0.000468 | 0.00207 | 0.000468 |
| Actinobacteria | 0.32 | 0.103293 | 0.261219 | 0.339 | 0.186275 | 0.147173 | 0.105434 |
| Armatimonadetes | 0.000656 | 0 | 0 | 0 | 0 | 0.00037 | 0 |
| Bacteroidetes | 50.2 | 22.65034 | 54.66715 | 59.1 | 52.08114 | 57.7147 | 60.50775 |
| Candidatus saccharibacteria | 0 | 3.98026 | 0 | 0 | 0 | 0 | 0 |
| Chlamydiae | 0.00198 | 0.015672 | 0 | 0.00177 | 0 | 0 | 0 |
| Chlorobi | 0 | 0 | 0 | 0.000384 | 0 | 0.000478 | 0 |
| Chloroflexi | 0.0302 | 0 | 0.000828 | 0.00137 | 0.000579 | 0.000718 | 0.000669 |
| Cyanobacteria | 0.455 | 0.021241 | 0.31165 | 0.326 | 0.267553 | 0.674151 | 0.253012 |
| Deferribacteres | 0.0811 | 0.024929 | 0.090076 | 0.294 | 0.16397 | 0.218214 | 0.377722 |
| Elusimicrobia | 0.0198 | 0.002234 | 0.003629 | 0.0543 | 0.044189 | 0.015803 | 0.023574 |
| Euryarchaeota | 0.00125 | 0.001119 | 0.086129 | 0.00333 | 0 | 0.00116 | 0.003897 |
| FBP | 0 | 0 | 0 | 0 | 0 | 0 | 0.000468 |
| Fibrobacteres | 0.291 | 0.002337 | 0 | 0 | 0 | 0 | 0 |
| Firmicutes | 44.1 | 54.5598 | 41.36305 | 28.2 | 33.43791 | 27.7715 | 28.81801 |
| Fusobacteria | 0.00217 | 0.000641 | 0 | 0.00101 | 0.017543 | 0.007304 | 0.000937 |
| Gemmatimonadetes | 0.0295 | 0 | 0 | 0.000384 | 0.000884 | 0 | 0.000535 |
| Lentisphaerae | 0.0136 | 0.000703 | 0.000827 | 0.0238 | 0.035973 | 0.063793 | 0.006504 |
| Nitrospirae | 0.00524 | 0 | 0 | 0.000443 | 0 | 0.000425 | 0 |
| Planctomycetes | 0.053 | 0.001007 | 0.001655 | 0.0347 | 0.04282 | 0.042728 | 0.015139 |
| Proteobacteria | 3.19 | 6.727346 | 1.735814 | 6.01 | 12.17655 | 12.5466 | 8.233009 |
| Spirochaetes | 0.338 | 11.76734 | 1.077 | 4.4 | 0.486021 | 0.123046 | 1.132232 |
| Synergistetes | 0 | 0.000661 | 0 | 0 | 0 | 0 | 0 |
| Tenericutes | 0.121 | 0.011443 | 0.033121 | 0.0331 | 0.045101 | 0.018365 | 0.119577 |
| TM7 | 0.403 | 0 | 0.366204 | 0.484 | 0.998149 | 0.634511 | 0.39663 |
| Verrucomicrobia | 0.0539 | 0.008913 | 0.001655 | 0.673 | 0.014872 | 0.016884 | 0.004429 |
| WS2 | 0.000656 | 0 | 0 | 0 | 0 | 0 | 0 |
| WS3 | 0.0059 | 0 | 0 | 0 | 0 | 0 | 0 |

NCD, normal chow diet; NCD-T, normal chow diet + atorvastatin treatment; HFD, high-fat diet; 5 mg/kg, HFD + 5mg/kg atorvastatin; 10 mg/kg, HFD + 10 mg/kg atorvastatin; 15 mg/kg, HFD + 15 mg/kg atorvastatin; 20 mg/kg, HFD + 20 mg/kg atorvastatin.

**Supplementary Table 2:** Relative abundance expressed in percentage of the identified families in this study.

| **Families** | **NCD** | **NCD-T** | **HFD** | **5 mg/kg** | **15 mg/kg** | **10 mg/kg** | **20 mg/kg** |
| --- | --- | --- | --- | --- | --- | --- | --- |
| Acaryochloridaceae | 0 | 0 | 0 | 0 | 0 | 0 | 0.000669 |
| Acetobacteraceae | 0 | 0.000589 | 0 | 0 | 0 | 0 | 0 |
| Acidaminobacteraceae | 0 | 0 | 0 | 0.000443 | 0 | 0 | 0.000468 |
| Acidaminococcaceae | 0 | 0.004993 | 0 | 0 | 0 | 0 | 0 |
| Acidithiobacillaceae | 0 | 0.000369 | 0 | 0 | 0 | 0 | 0 |
| Acidobacteriaceae | 0 | 0.000156 | 0 | 0 | 0 | 0 | 0 |
| Actinomycetaceae | 0 | 0 | 0 | 0 | 0.000468 | 0 | 0.000875 |
| Actinosynnemataceae | 0.0059 | 0 | 0 | 0 | 0 | 0 | 0 |
| Aerococcaceae | 0.198 | 0.003713 | 0.278119 | 0.55 | 0.294074 | 0.250885 | 0.356878 |
| Aeromonadaceae | 0 | 0.00016 | 0 | 0 | 0 | 0 | 0 |
| Alcaligenaceae | 0.0889 | 0.002693 | 0.131538 | 1.76 | 2.426847 | 1.67112 | 0.568191 |
| Alcanivoracaceae | 0 | 0 | 0 | 0 | 0 | 0.000407 | 0 |
| Alicyclobacillaceae | 0 | 0.002987 | 0 | 0 | 0 | 0 | 0 |
| Alteromonadaceae | 0 | 0 | 0 | 0 | 0.00149 | 0 | 0 |
| Anaeroplasmataceae | 0.0664 | 0.002747 | 0.006349 | 0.00667 | 0.015627 | 0.004494 | 0.002141 |
| Anaplasmataceae | 0 | 0.000152 | 0 | 0 | 0 | 0 | 0 |
| Bacillaceae | 0.014 | 0.042518 | 0.005945 | 0.00159 | 0.002069 | 0.002391 | 0 |
| Bacillales | 0 | 0.007844 | 0 | 0 | 0 | 0 | 0 |
| Bacteria | 0 | 0.000624 | 0 | 0 | 0 | 0 | 0 |
| Bacteroidaceae | 7.02 | 4.461195 | 7.895991 | 9.71 | 9.950795 | 13.58467 | 8.701904 |
| Balneolaceae | 0.000631 | 0 | 0 | 0 | 0 | 0 | 0 |
| Barnesiellaceae | 0.00227 | 0 | 0.118846 | 0.00146 | 0.003866 | 0.001948 | 0.004421 |
| Beijerinckiaceae | 0.00262 | 0.00016 | 0 | 0 | 0 | 0 | 0 |
| Bifidobacteriaceae | 0.0377 | 0.028527 | 0.017076 | 0.046 | 0.031564 | 0.019626 | 0.01533 |
| Brachyspiraceae | 0.054 | 0.351766 | 0.012377 | 0.0439 | 0.048479 | 0.025796 | 0.143936 |
| Bradyrhizobiaceae | 0.0131 | 0 | 0 | 0 | 0 | 0 | 0 |
| Brevibacteriaceae | 0 | 0 | 0 | 0 | 0 | 0 | 0 |
| Brucellaceae | 0 | 0 | 0 | 0 | 0.000579 | 0 | 0 |
| Burkholderiaceae | 0 | 0.001011 | 0 | 0 | 0 | 0 | 0 |
| Burkholderiales | 0 | 0 | 0 | 0 | 0 | 0 | 0 |
| C111 | 0.00721 | 0 | 0.000827 | 0 | 0 | 0 | 0 |
| Caldilineaceae | 0 | 0 | 0 | 0 | 0.000579 | 0 | 0 |
| Caldithrixaceae | 0 | 0 | 0 | 0 | 0 | 0 | 0 |
| Campylobacteraceae | 0.028 | 0.004695 | 0.005197 | 0.0205 | 0 | 0 | 0.014314 |
| Candidatus midichloriaceae | 0 | 0.012646 | 0 | 0 | 0 | 0 | 0 |
| Candidatus saccharibacteria | 0 | 3.98026 | 0 | 0 | 0 | 0 | 0 |
| Carnobacteriaceae | 0.00317 | 0.007884 | 0.002229 | 0.00724 | 0.003141 | 0.002603 | 0.003292 |
| Catabacteriaceae | 0 | 0.002799 | 0 | 0 | 0 | 0 | 0 |
| Caulobacteraceae | 0.00721 | 0 | 0 | 0.00159 | 0 | 0 | 0 |
| Cellulomonadaceae | 0.000656 | 0 | 0 | 0 | 0 | 0 | 0 |
| Cerasicoccaceae | 0 | 0 | 0 | 0.00334 | 0 | 0 | 0 |
| Chitinophagaceae | 0.0243 | 0 | 0 | 0 | 0.000941 | 0 | 0 |
| Chlamydiaceae | 0.00198 | 0.015672 | 0 | 0.00177 | 0 | 0 | 0 |
| Christensenellaceae | 0.0726 | 0.006854 | 0.008765 | 0.0386 | 0.072445 | 0.044753 | 0.030984 |
| Chromatiaceae | 0.000656 | 0 | 0 | 0.000357 | 0 | 0 | 0 |
| Chromobacteriaceae | 0 | 0 | 0 | 0 | 0 | 0 | 0 |
| Chthoniobacteraceae | 0.0164 | 0 | 0 | 0 | 0 | 0 | 0 |
| Clostridiaceae | 1.78 | 19.3727 | 6.104721 | 2.41 | 2.36311 | 2.592102 | 2.822618 |
| Clostridiales | 0 | 1.68921 | 0 | 0 | 0 | 0 | 0 |
| Clostridiales family xi. Incertae sedis | 0 | 0.009268 | 0 | 0 | 0 | 0 | 0 |
| Clostridiales family xiii. Incertae sedis | 0 | 0.025207 | 0 | 0 | 0 | 0 | 0 |
| Cohaesibacteraceae | 0 | 0 | 0 | 0.000443 | 0 | 0 | 0 |
| Comamonadaceae | 0.00524 | 0 | 0 | 0.0025 | 0.000579 | 0.002629 | 0 |
| Coriobacteriaceae | 0.0081 | 0.025553 | 0.04079 | 0.0513 | 0.045043 | 0.027908 | 0.038726 |
| Corynebacteriaceae | 0.0646 | 0.042065 | 0.103911 | 0.224 | 0.096453 | 0.094038 | 0.04199 |
| Coxiellaceae | 0 | 0 | 0 | 0 | 0 | 0 | 0 |
| Cryomorphaceae | 0.000656 | 2.735793 | 0 | 0 | 0 | 0 | 0 |
| Cystobacteraceae | 0.000656 | 0 | 0 | 0 | 0 | 0 | 0 |
| Cytophagaceae | 0.00131 | 0.246942 | 0 | 0 | 0.000468 | 0 | 0 |
| Deferribacteraceae | 0.0811 | 0.024929 | 0.090076 | 0.294 | 0.16397 | 0.218214 | 0.377722 |
| Dehalobacteriaceae | 0.0142 | 0 | 0.04158 | 0.0032 | 0.006728 | 0.012237 | 0.007166 |
| Dermabacteraceae | 0 | 0.001452 | 0 | 0 | 0 | 0 | 0 |
| Desulfobacteraceae | 0.000631 | 0 | 0 | 0.000886 | 0 | 0.00037 | 0 |
| Desulfovibrionaceae | 1.39 | 3.466387 | 0.553811 | 2.21 | 5.759076 | 5.558006 | 2.289563 |
| EB1017 | 0.00262 | 0 | 0 | 0 | 0 | 0 | 0 |
| Ellin517 | 0 | 0 | 0 | 0 | 0 | 0.00037 | 0 |
| Ellin6075 | 0.0716 | 0 | 0 | 0 | 0 | 0 | 0 |
| Elusimicrobiaceae | 0.019 | 0.002234 | 0.003629 | 0.0543 | 0.044189 | 0.015803 | 0.023574 |
| Enterobacteriaceae | 0.0244 | 0.062772 | 0.015517 | 0.0268 | 0.03041 | 0.024789 | 0.027167 |
| Enterococcaceae | 0.00679 | 0.006367 | 0.001318 | 0.00753 | 0.008681 | 0 | 0.004167 |
| Erysipelotrichaceae | 0.021 | 1.45582 | 0.014939 | 0.0808 | 0.029048 | 0.02992 | 0.07821 |
| Erythrobacteraceae | 0.00328 | 0 | 0 | 0 | 0 | 0 | 0 |
| Eubacteriaceae | 0.000656 | 4.100831 | 0 | 0 | 0 | 0 | 0 |
| Eukaryota | 0 | 0 | 0 | 0 | 0 | 0 | 0 |
| Euzebyaceae | 0.00656 | 0 | 0 | 0 | 0 | 0 | 0 |
| F16 | 0.4 | 0 | 0.366204 | 0.484 | 0.99757 | 0.634033 | 0.39663 |
| Fibrobacteraceae | 0.29 | 0.002337 | 0 | 0 | 0 | 0 | 0 |
| Fimbriimonadaceae | 0.000656 | 0 | 0 | 0 | 0 | 0 | 0 |
| Flammeovirgaceae | 0 | 0.001769 | 0 | 0 | 0 | 0 | 0 |
| Flavobacteriaceae | 0 | 0.062255 | 0 | 0.000443 | 0.000468 | 0 | 0.002624 |
| Frankiaceae | 0.000656 | 0 | 0 | 0 | 0 | 0 | 0 |
| Fusobacteriaceae | 0.00217 | 0.000641 | 0 | 0.00101 | 0.017543 | 0.007304 | 0.000937 |
| Gaiellaceae | 0.0184 | 0 | 0 | 0 | 0.001012 | 0 | 0 |
| Gammaproteobacteria | 0 | 0.000193 | 0 | 0 | 0 | 0 | 0 |
| Gemellaceae | 0 | 0 | 0.002146 | 0.00151 | 0.003035 | 0.008547 | 0.000535 |
| Gemmataceae | 0.00262 | 0 | 0.001655 | 0 | 0 | 0 | 0 |
| Gemmatimonadaceae | 0.00197 | 0 | 0 | 0 | 0 | 0 | 0 |
| Geobacteraceae | 0 | 0.000975 | 0 | 0 | 0 | 0 | 0 |
| Geodermatophilaceae | 0.00918 | 0 | 0 | 0 | 0 | 0.000478 | 0 |
| Gloeobacterales | 0 | 0.021241 | 0 | 0 | 0 | 0 | 0 |
| Gracilibacteraceae | 0 | 0.008368 | 0 | 0 | 0 | 0 | 0 |
| Halanaerobiaceae | 0 | 0 | 0 | 0 | 0 | 0 | 0.000875 |
| Halomonadaceae | 0 | 0 | 0 | 0.000443 | 0.000468 | 0 | 0.000875 |
| Haloplasmataceae | 0 | 0.000196 | 0 | 0 | 0 | 0 | 0 |
| Helicobacteraceae | 1.09 | 2.94895 | 0.952062 | 1.81 | 3.812872 | 5.046398 | 5.169289 |
| Holophagaceae | 0 | 0 | 0 | 0 | 0 | 0 | 0 |
| Hyphomicrobiaceae | 0.0229 | 0.003803 | 0 | 0 | 0.00152 | 0 | 0.000669 |
| Hyphomonadaceae | 0.00197 | 0.000714 | 0 | 0 | 0 | 0 | 0 |
| Hypotrichomonadidae | 0 | 0.06218 | 0 | 0 | 0 | 0 | 0 |
| Iamiaceae | 0.000656 | 0 | 0 | 0 | 0 | 0 | 0 |
| Idiomarinaceae | 0 | 0 | 0 | 0 | 0 | 0 | 0.000875 |
| Ignavibacteriaceae | 0 | 0 | 0 | 0.000384 | 0 | 0.000478 | 0 |
| Isosphaeraceae | 0 | 0 | 0 | 0 | 0 | 0.00037 | 0 |
| Kineosporiaceae | 0.00197 | 0 | 0 | 0 | 0 | 0 | 0 |
| koll13 | 0 | 0 | 0 | 0.000443 | 0.000936 | 0 | 0 |
| Kopriimonadaceae | 0 | 0.031419 | 0 | 0 | 0 | 0 | 0 |
| Koribacteraceae | 0.00131 | 0 | 0 | 0 | 0 | 0 | 0 |
| Kouleothrixaceae | 0.00262 | 0 | 0 | 0 | 0 | 0 | 0 |
| Labridae | 0 | 0.023949 | 0 | 0 | 0 | 0 | 0 |
| Lachnospiraceae | 3.51 | 15.75242 | 3.409313 | 1.86 | 1.81833 | 1.354114 | 1.632785 |
| Lactobacillaceae | 0.388 | 2.41151 | 1.248502 | 0.908 | 1.285398 | 0.342007 | 1.043458 |
| Leptotrichiaceae | 0 | 0 | 0 | 0 | 0 | 0 | 0 |
| Leuconostocaceae | 0.00135 | 0.025449 | 0 | 0.00259 | 0.003473 | 0.000849 | 0.003342 |
| Listeriaceae | 0 | 0.001412 | 0 | 0 | 0 | 0 | 0 |
| Marinicellaceae | 0 | 0 | 0 | 0.000443 | 0 | 0.00037 | 0 |
| Marinifilaceae | 0 | 0 | 0 | 0 | 0 | 0 | 0 |
| Marinilabiliaceae | 0 | 0.063366 | 0 | 0 | 0 | 0 | 0 |
| mb2424 | 0.00918 | 0 | 0 | 0 | 0.000468 | 0 | 0 |
| Methanobacteriaceae | 0.000631 | 0.001119 | 0.086129 | 0.00232 | 0 | 0 | 0.001074 |
| Methanomassiliicoccaceae | 0.000617 | 0 | 0 | 0.00101 | 0 | 0.00116 | 0.002823 |
| Methylocystaceae | 0 | 0 | 0 | 0 | 0 | 0.000957 | 0 |
| Microbacteriaceae | 0 | 0 | 0.000742 | 0.000531 | 0 | 0 | 0 |
| Micrococcaceae | 0.0321 | 0.002487 | 0.00157 | 0.000357 | 0.001047 | 0 | 0.000468 |
| Micromonosporaceae | 0.00325 | 0 | 0 | 0 | 0 | 0 | 0 |
| mitochondria | 0 | 0 | 0 | 0 | 0 | 0.000478 | 0 |
| Mogibacteriaceae | 0.0933 | 0 | 0.295507 | 0.148 | 0.25455 | 0.214275 | 0.176522 |
| Moraxellaceae | 0.0332 | 0.000328 | 0.043085 | 0.0503 | 0.060728 | 0.076915 | 0.007757 |
| MVS-65 | 0.000656 | 0 | 0 | 0 | 0 | 0 | 0 |
| Mycobacteriaceae | 0.00197 | 0 | 0 | 0 | 0 | 0 | 0 |
| Mycoplasmataceae | 0.0187 | 0.007705 | 0.009652 | 0.0099 | 0 | 0 | 0.102479 |
| Myxococcaceae | 0.00131 | 0 | 0 | 0 | 0 | 0 | 0 |
| NB1-i | 0.000656 | 0 | 0 | 0 | 0 | 0 | 0 |
| Neisseriaceae | 0.000631 | 0 | 0 | 0 | 0 | 0 | 0.000468 |
| Nitrosomonadaceae | 0.000656 | 0 | 0 | 0 | 0 | 0 | 0 |
| Nitrospiraceae | 0.00459 | 0 | 0 | 0.000443 | 0 | 0 | 0 |
| Nocardiaceae | 0.00131 | 0.000995 | 0.000827 | 0.00107 | 0 | 0 | 0 |
| Nocardioidaceae | 0.00852 | 0 | 0 | 0 | 0 | 0 | 0 |
| Oceanospirillaceae | 0.00316 | 0 | 0 | 0.00354 | 0.001515 | 0.000778 | 0.007534 |
| Odoribacteraceae | 1.13 | 0 | 0.514731 | 0.656 | 0.297877 | 0.212337 | 0.516383 |
| Oligosphaeraceae | 0 | 0.000349 | 0 | 0 | 0 | 0 | 0 |
| OM27 | 0.000656 | 0 | 0 | 0 | 0 | 0 | 0 |
| Opitutaceae | 0 | 0.000321 | 0 | 0 | 0 | 0 | 0 |
| Oscillatoriales | 0 | 0 | 0 | 0 | 0 | 0 | 0 |
| Oscillospiraceae | 0 | 0.025415 | 0 | 0 | 0 | 0 | 0 |
| Oxalobacteraceae | 0.00291 | 0 | 0 | 0 | 0 | 0.000478 | 0 |
| Paenibacillaceae | 0.000656 | 0.000156 | 0 | 0.000531 | 0.000941 | 0 | 0 |
| Paraprevotellaceae | 8.17 | 0 | 5.769641 | 7.9 | 10.34079 | 13.41867 | 6.99998 |
| Parvularculaceae | 0 | 0.001315 | 0 | 0 | 0 | 0 | 0 |
| Pasteurellaceae | 0.00765 | 0.005658 | 0.007671 | 0.00696 | 0.003035 | 0.004599 | 0.001071 |
| Patulibacteraceae | 0.00262 | 0 | 0 | 0 | 0 | 0 | 0 |
| Peptococcaceae | 0.0684 | 0.079952 | 0.026723 | 0.166 | 0.209747 | 0.345644 | 0.119613 |
| Peptoniphilaceae | 0 | 0.000152 | 0 | 0 | 0 | 0 | 0 |
| Peptostreptococcaceae | 1.32 | 0.005522 | 4.448755 | 1.68 | 1.928081 | 2.135429 | 2.306793 |
| Phyllobacteriaceae | 0.00721 | 0 | 0 | 0 | 0 | 0 | 0 |
| Pirellulaceae | 0.0196 | 0 | 0 | 0.0344 | 0.04282 | 0.042358 | 0.015139 |
| Piscirickettsiaceae | 0 | 0 | 0 | 0 | 0 | 0 | 0.001343 |
| Planctomycetaceae | 0.00131 | 0.001007 | 0 | 0 | 0 | 0 | 0 |
| Planococcaceae | 0 | 0.001693 | 0.003955 | 0.00493 | 0.002673 | 0.000778 | 0.001004 |
| Porphyromonadaceae | 1.41 | 9.817094 | 0.355338 | 1.61 | 1.482441 | 1.629801 | 1.616789 |
| Prevotellaceae | 18.2 | 2.982166 | 18.91087 | 22.8 | 10.48254 | 9.020469 | 21.06698 |
| Prolixibacteraceae | 0 | 0.00346 | 0 | 0 | 0 | 0 | 0 |
| Propionibacteriaceae | 0.00328 | 0 | 0 | 0.000384 | 0.000468 | 0.000478 | 0.000537 |
| PRR-10 | 0.00393 | 0 | 0 | 0 | 0 | 0 | 0 |
| Pseudoalteromonadaceae | 0.00126 | 0 | 0 | 0.00221 | 0.00515 | 0.00037 | 0.001749 |
| Pseudomonadaceae | 0.00406 | 0.000672 | 0.001318 | 0.00278 | 0.003915 | 0.004179 | 0.004155 |
| Pseudonocardiaceae | 0.0151 | 0 | 0.000828 | 0 | 0.000884 | 0 | 0 |
| R4-45B | 0.0108 | 0 | 0 | 0.0203 | 0.033623 | 0.05493 | 0 |
| RB40 | 0.00469 | 0 | 0 | 0 | 0 | 0 | 0 |
| RF16 | 0.506 | 0 | 0 | 0.59 | 0.296056 | 0.327205 | 0.108547 |
| RFP12 | 0.00733 | 0 | 0 | 0.0114 | 0.012371 | 0.014451 | 0 |
| Rhizobiaceae | 0.00738 | 0.000156 | 0.002227 | 0.0016 | 0.000941 | 0.002056 | 0 |
| Rhodobacteraceae | 0.00323 | 0.001334 | 0 | 0.00142 | 0.004189 | 0 | 0.001606 |
| Rhodobiaceae | 0.0354 | 0.076977 | 0 | 0 | 0 | 0 | 0.000468 |
| Rhodocyclaceae | 0 | 0.000165 | 0 | 0 | 0 | 0 | 0 |
| Rhodospirillaceae | 0.00787 | 0.00512 | 0 | 0.000443 | 0.000579 | 0 | 0.000468 |
| Rickettsiaceae | 0 | 0 | 0 | 0 | 0 | 0 | 0 |
| Rickettsiales | 0 | 0 | 0 | 0 | 0 | 0 | 0 |
| Rikenellaceae | 0.839 | 2.041225 | 1.518574 | 0.251 | 0.762476 | 1.006466 | 0.985351 |
| Rubrobacteraceae | 0.00918 | 0.002213 | 0 | 0.000357 | 0.001591 | 0.000478 | 0 |
| Ruminococcaceae | 12.6 | 6.550595 | 7.638437 | 9.47 | 13.70623 | 11.90085 | 10.03305 |
| S24-7 | 9.12 | 0 | 15.09036 | 11.8 | 15.17122 | 13.4277 | 15.83904 |
| Saprospiraceae | 0.000656 | 0 | 0 | 0.000357 | 0 | 0 | 0 |
| Shewanellaceae | 0 | 0 | 0 | 0 | 0 | 0.000425 | 0 |
| Sinobacteraceae | 0.00656 | 0 | 0 | 0 | 0.000579 | 0 | 0.001137 |
| Solibacteraceae | 0.00197 | 0 | 0 | 0 | 0 | 0 | 0 |
| Solirubrobacteraceae | 0.0059 | 0 | 0 | 0 | 0 | 0 | 0 |
| Sphingobacteriaceae | 0.00225 | 0.235071 | 0 | 0.00266 | 0.003536 | 0.001913 | 0 |
| Sphingomonadaceae | 0.231 | 0.00016 | 0.000828 | 0.00179 | 0.003505 | 0.00433 | 0 |
| Spirochaetaceae | 0.284 | 11.41558 | 1.064623 | 4.36 | 0.437542 | 0.09725 | 0.988296 |
| Spiroplasmataceae | 0 | 0.000991 | 0 | 0 | 0 | 0 | 0 |
| Sporichthyaceae | 0.00131 | 0 | 0 | 0 | 0 | 0 | 0 |
| Sporolactobacillaceae | 0 | 0.000156 | 0 | 0 | 0 | 0 | 0 |
| Staphylococcaceae | 0.166 | 0.030917 | 0.209317 | 0.0907 | 0.066245 | 0.034751 | 0.053449 |
| Streptococcaceae | 0.0778 | 0.600965 | 0.058735 | 0.0797 | 0.098555 | 0.030216 | 0.057814 |
| Streptomycetaceae | 0.00197 | 0 | 0 | 0 | 0 | 0 | 0 |
| Succinivibrionaceae | 0 | 0.047025 | 0 | 8.00E-04 | 0 | 0.000956 | 0 |
| Sutterellaceae | 0 | 0.043545 | 0 | 0 | 0 | 0 | 0 |
| Symbiobacteriaceae | 0 | 0.000328 | 0 | 0 | 0 | 0 | 0 |
| Synergistaceae | 0 | 0.000661 | 0 | 0 | 0 | 0 | 0 |
| Syntrophobacteraceae | 0.00918 | 0 | 0 | 0.00042 | 0 | 0 | 0 |
| Syntrophomonadaceae | 0 | 0.000156 | 0 | 0 | 0 | 0 | 0 |
| Thermoactinomycetaceae | 0 | 0 | 0 | 0 | 0 | 0 | 0 |
| Thermoanaerobacteraceae | 0 | 0 | 0 | 0 | 0 | 0 | 0 |
| Thermodesulfovibrionaceae | 0 | 0 | 0 | 0 | 0 | 0.000425 | 0 |
| Thermoplasmataceae | 0 | 0 | 0 | 0 | 0 | 0 | 0 |
| Thiotrichaceae | 0.000631 | 0.00175 | 0 | 0.000443 | 0 | 0 | 0.000875 |
| Trichomonadidae | 0 | 0.007349 | 0 | 0 | 0 | 0 | 0 |
| Tritrichomonadidae | 0 | 0.026266 | 0 | 0 | 0 | 0 | 0 |
| Turicibacteraceae | 1.47 | 0 | 1.781808 | 0.788 | 0.054699 | 0.114189 | 0.475897 |
| Unclassified | 26.3868 | 0 | 19.91101 | 13.69173 | 14.00855 | 13.5785 | 14.17728 |
| Veillonellaceae | 0.578 | 2.325633 | 0.70444 | 0.396 | 0.86006 | 0.682755 | 0.500223 |
| Verrucomicrobiaceae | 0.0301 | 0.008592 | 0.001655 | 0.658 | 0.002502 | 0.001692 | 0.004429 |
| Vibrionaceae | 0 | 0.001027 | 0 | 0 | 0 | 0 | 0 |
| Victivallaceae | 0.00288 | 0.000354 | 0.000827 | 0.00353 | 0.002349 | 0.008863 | 0.006504 |
| Weeksellaceae | 0.000677 | 0 | 0 | 0.00196 | 0.000884 | 0.000956 | 0.001337 |
| Xanthomonadaceae | 0.021 | 0.004424 | 0.011736 | 0 | 0.001896 | 0.014243 | 0 |
| Xenococcaceae | 0 | 0 | 0 | 0 | 0 | 0.000478 | 0 |
| Yaniellaceae | 0.00518 | 0 | 0.094647 | 0.0141 | 0.006809 | 0.003742 | 0.00637 |
| 0319-6A21 | 0.000656 | 0 | 0 | 0 | 0 | 0 | 0 |
| A4b | 0.000656 | 0 | 0 | 0 | 0 | 0 | 0 |

NCD, normal chow diet; NCD-T, normal chow diet + atorvastatin treatment; HFD, high-fat diet; 5 mg/kg, HFD + 5mg/kg atorvastatin; 10 mg/kg, HFD + 10 mg/kg atorvastatin; 15 mg/kg, HFD + 15 mg/kg atorvastatin; 20 mg/kg, HFD + 20 mg/kg atorvastatin.

**Supplementary Table 3: Relative abundance expressed in percentage of the identified genera in this study.**

| **Genera** | **NCD** | **NCD-T** | **HFD** | **5 mg/kg** | **15 mg/kg** | **10 mg/kg** | **20 mg/kg** |
| --- | --- | --- | --- | --- | --- | --- | --- |
| *Abiotrophia* | 0 | 0.000925 | 0 | 0 | 0 | 0 | 0 |
| *Acetanaerobacterium* | 0 | 0.012674 | 0 | 0 | 0 | 0 | 0 |
| *Acetitomaculum* | 0 | 0.00118 | 0 | 0 | 0 | 0 | 0 |
| *Acetivibrio* | 0 | 0.105434 | 0 | 0 | 0 | 0 | 0 |
| *Acidaminococcus* | 0.001308 | 0.001151 | 0 | 0.000714 | 0 | 0.001047 | 0 |
| *Acidithiobacillus* | 0 | 0.000369 | 0 | 0 | 0 | 0 | 0 |
| *Acidobacterium* | 0 | 0.000156 | 0 | 0 | 0 | 0 | 0 |
| *Acinetobacter* | 0.0059 | 0.000328 | 0.000742 | 0.002221 | 0.000957 | 0 | 0 |
| *Actinobacillus* | 0 | 0.000477 | 0 | 0 | 0 | 0 | 0 |
| *Actinomyces* | 0 | 0 | 0 | 0 | 0 | 0.000468 | 0.000875 |
| *Adhaeribacter* | 0 | 0.059948 | 0 | 0 | 0 | 0 | 0 |
| *Adlercreutzia* | 0.000617 | 0.003544 | 0.011988 | 0.014567 | 0.011546 | 0.016438 | 0.012455 |
| *Aerococcus* | 0.061605 | 0.000468 | 0.147034 | 0.335768 | 0.144427 | 0.158358 | 0.278751 |
| *Aeromicrobium* | 0.002622 | 0 | 0 | 0 | 0 | 0 | 0 |
| *Aeromonas* | 0 | 0.00016 | 0 | 0 | 0 | 0 | 0 |
| *Afifella* | 0.035402 | 0 | 0 | 0 | 0 | 0 | 0.000468 |
| *Aggregatibacter* | 0.00275 | 0 | 0.002061 | 0.001863 | 0.002592 | 0.003035 | 0 |
| *Agrobacterium* | 0.000656 | 0 | 0.000742 | 0 | 0 | 0 | 0 |
| *Akkermansia* | 0.026855 | 0.008592 | 0.001655 | 0.658189 | 0.001692 | 0.002502 | 0.004429 |
| *Alcaligenes* | 0 | 0.000477 | 0 | 0 | 0.001435 | 0 | 0 |
| *Alcanivorax* | 0 | 0 | 0 | 0 | 0.000407 | 0 | 0 |
| *Alicyclobacillus* | 0 | 0.002987 | 0 | 0 | 0 | 0 | 0 |
| *Alistipes* | 0.020054 | 0.41052 | 0.017659 | 0.009812 | 0.014814 | 0.007789 | 0.012653 |
| *Alkalibacter* | 0 | 0.005229 | 0 | 0 | 0 | 0 | 0 |
| *Alkaliflexus* | 0 | 0.001844 | 0 | 0 | 0 | 0 | 0 |
| *Alkaliphilus* | 0 | 0.003157 | 0 | 0 | 0 | 0 | 0 |
| *Allisonella* | 0 | 0.000333 | 0 | 0 | 0 | 0 | 0 |
| *Allobaculum* | 0.000631 | 0 | 0.006516 | 0.003699 | 0.001453 | 0.003009 | 0.003886 |
| *Alloprevotella* | 0 | 0.424007 | 0 | 0 | 0 | 0 | 0 |
| *Anaerobacterium* | 0 | 0.000525 | 0 | 0 | 0 | 0 | 0 |
| *Anaerobiospirillum* | 0 | 0.034474 | 0 | 0 | 0 | 0 | 0 |
| *Anaerofilum* | 0 | 0.006922 | 0 | 0 | 0 | 0 | 0 |
| *Anaerofustis* | 0.000656 | 0.000345 | 0 | 0 | 0 | 0 | 0 |
| *Anaeromyxobacter* | 0.001311 | 0 | 0 | 0 | 0 | 0 | 0 |
| *Anaerophaga* | 0 | 0.005001 | 0 | 0 | 0 | 0 | 0 |
| *Anaeroplasma* | 0.06644 | 0.002422 | 0.006349 | 0.006316 | 0.004494 | 0.015627 | 0.002141 |
| *Anaerorhabdus* | 0 | 0.000357 | 0 | 0 | 0 | 0 | 0 |
| *Anaerosporobacter* | 0 | 0.026284 | 0 | 0 | 0 | 0 | 0 |
| *Anaerostipes* | 0.033331 | 0.152819 | 0.010564 | 0.005976 | 0.000425 | 0.005079 | 0.017656 |
| *Anaerotruncus* | 0 | 0.075994 | 0.001655 | 0 | 0 | 0 | 0.002211 |
| *Anaerovibrio* | 0.167088 | 0.34194 | 0.690486 | 0.15926 | 0.124924 | 0.823137 | 0.219385 |
| *Anaerovorax* | 0 | 0.024903 | 0 | 0 | 0 | 0 | 0 |
| *Anoxybacillus* | 0 | 0.002296 | 0 | 0 | 0 | 0 | 0 |
| *Arcobacter* | 0 | 0.000473 | 0 | 0 | 0 | 0 | 0 |
| *Arenibacter* | 0 | 0.000316 | 0 | 0 | 0 | 0 | 0 |
| *Arthrobacter* | 0.020324 | 0 | 0 | 0 | 0 | 0 | 0.000468 |
| *Asaccharospora* | 0 | 0.00039 | 0 | 0 | 0 | 0 | 0 |
| *Asteroleplasma* | 0 | 0.000325 | 0 | 0 | 0 | 0 | 0 |
| *Atopobium* | 0.000631 | 0.00121 | 0 | 0 | 0 | 0 | 0 |
| *Atopostipes* | 0 | 0.0054 | 0 | 0 | 0 | 0 | 0 |
| *Aureibacter* | 0 | 0.000866 | 0 | 0 | 0 | 0 | 0 |
| *Azospirillum* | 0 | 0.002902 | 0 | 0 | 0 | 0 | 0 |
| *Bacillus* | 0.013399 | 0.040223 | 0.002312 | 0.001594 | 0.002391 | 0.000579 | 0 |
| *Bacteroides* | 7.016214 | 4.460838 | 7.895248 | 9.714496 | 13.58345 | 9.950795 | 8.701367 |
| *Balneimonas* | 0.000656 | 0 | 0 | 0 | 0 | 0 | 0 |
| *Barnesiella* | 0 | 5.087462 | 0 | 0 | 0 | 0 | 0 |
| *Bifidobacterium* | 0.037691 | 0.028527 | 0.017076 | 0.045983 | 0.019626 | 0.031564 | 0.01533 |
| *Bilophila* | 0.019492 | 0.049337 | 0.002398 | 0.029478 | 0.070113 | 0.105074 | 0.057135 |
| *Blastochloris* | 0 | 0.000476 | 0 | 0 | 0 | 0 | 0 |
| *Blautia* | 0.032424 | 0.54617 | 0.021115 | 0.022239 | 0.020353 | 0.045574 | 0.014859 |
| *Brachybacterium* | 0 | 0.001452 | 0 | 0 | 0 | 0 | 0 |
| *Brachyspira* | 0.054032 | 0.351766 | 0.012377 | 0.043945 | 0.025796 | 0.048479 | 0.143936 |
| *Bradyrhizobium* | 0.009834 | 0 | 0 | 0 | 0 | 0 | 0 |
| *Brevundimonas* | 0 | 0 | 0 | 0.000531 | 0 | 0 | 0 |
| *Bulleidia* | 0 | 0 | 0 | 0 | 0 | 0 | 0.000875 |
| *Butyricicoccus* | 0 | 0.014342 | 0 | 0 | 0 | 0 | 0 |
| *Butyricimonas* | 0.037307 | 0.009519 | 0.022991 | 0.02176 | 0.048595 | 0.024657 | 0.031592 |
| *Butyrivibrio* | 0 | 0.055685 | 0 | 0 | 0 | 0 | 0 |
| *Campylobacter* | 0.027958 | 0.004222 | 0.005197 | 0.020491 | 0 | 0 | 0.014314 |
| *Candidatus Arthromitus* | 0.005649 | 0 | 0.008273 | 0.005068 | 0.004186 | 0.001022 | 0.005888 |
| *Candidatus arthromitus* | 0 | 0.008888 | 0 | 0 | 0 | 0 | 0 |
| *Candidatus cyrtobacter* | 0 | 0.012646 | 0 | 0 | 0 | 0 | 0 |
| *Candidatus Koribacter* | 0.001311 | 0 | 0 | 0 | 0 | 0 | 0 |
| *Candidatus saccharimonas* | 0 | 3.98026 | 0 | 0 | 0 | 0 | 0 |
| *Candidatus soleaferrea* | 0 | 0.306317 | 0 | 0 | 0 | 0 | 0 |
| *Candidatus Solibacter* | 0.001967 | 0 | 0 | 0 | 0 | 0 | 0 |
| *Capnocytophaga* | 0 | 0.000513 | 0 | 0 | 0 | 0 | 0 |
| *Carnobacterium* | 0 | 0 | 0.000828 | 0.003105 | 0.002124 | 0 | 0 |
| *Catabacter* | 0 | 0.002799 | 0 | 0 | 0 | 0 | 0 |
| *Catenibacterium* | 0 | 0.002689 | 0 | 0 | 0 | 0.000468 | 0 |
| *Catonella* | 0 | 0.000703 | 0 | 0 | 0 | 0 | 0 |
| *Cellvibrio* | 0 | 0.000672 | 0 | 0 | 0 | 0 | 0 |
| CF231 | 4.873143 | 0 | 1.189436 | 2.760802 | 4.635618 | 4.315293 | 1.033848 |
| *Chlamydia* | 0.001985 | 0.015672 | 0 | 0.001771 | 0 | 0 | 0 |
| *Christensenella* | 0 | 0.006854 | 0 | 0 | 0 | 0 | 0 |
| *Chromohalobacter* | 0 | 0 | 0 | 0 | 0 | 0.000468 | 0.000875 |
| *Chryseobacterium* | 0.000677 | 0 | 0 | 0.00196 | 0.000956 | 0.000884 | 0.001337 |
| *Chthoniobacter* | 0.000656 | 0 | 0 | 0 | 0 | 0 | 0 |
| *Citrobacter* | 0 | 0 | 0 | 0.000886 | 0 | 0 | 0 |
| *Clostridiisalibacter* | 0 | 0 | 0 | 0 | 0 | 0.000468 | 0.000537 |
| *Clostridium* | 0.151631 | 19.31138 | 0.717711 | 0.193711 | 0.046055 | 0.077128 | 0.145841 |
| *Coccinistipes* | 0 | 0.000859 | 0 | 0 | 0 | 0 | 0 |
| *Coenonia* | 0 | 0.004136 | 0 | 0 | 0 | 0 | 0 |
| *Collinsella* | 0.001287 | 0.005438 | 0.012547 | 0.012222 | 0.006836 | 0.007142 | 0.012855 |
| *Coprobacillus* | 0 | 0 | 0 | 0.000531 | 0.001481 | 0 | 0 |
| *Coprobacter* | 0 | 0.004904 | 0 | 0 | 0 | 0 | 0 |
| *Coprococcus* | 0.39058 | 0.184205 | 0.985247 | 0.662656 | 0.410563 | 0.298087 | 0.224908 |
| *Corynebacterium* | 0.064588 | 0.042065 | 0.103911 | 0.223981 | 0.094038 | 0.096453 | 0.04199 |
| *Cupriavidus* | 0 | 0.000193 | 0 | 0 | 0.000478 | 0 | 0 |
| *Cytophaga* | 0 | 0.168534 | 0 | 0 | 0 | 0 | 0 |
| DA101 | 0.015734 | 0 | 0 | 0 | 0 | 0 | 0 |
| *Dehalobacterium* | 0.013525 | 0.006604 | 0.020693 | 0.000531 | 0.003395 | 0.004051 | 0.004353 |
| *Delftia* | 0 | 0 | 0 | 0.002143 | 0.002629 | 0.000579 | 0 |
| *Denitrobacterium* | 0 | 0.000345 | 0 | 0 | 0 | 0 | 0 |
| *Desulfococcus* | 0.000631 | 0 | 0 | 0.000443 | 0.00037 | 0 | 0 |
| *Desulfotomaculum* | 0 | 0.064467 | 0 | 0 | 0 | 0 | 0 |
| *Desulfovibrio* | 0.326407 | 3.416569 | 0.272135 | 0.165054 | 0.343894 | 0.69526 | 0.265469 |
| *Devosia* | 0.001311 | 0.003327 | 0 | 0 | 0 | 0 | 0 |
| *Dialister* | 0.006591 | 0.045603 | 0.00157 | 0.00441 | 0.004361 | 0.003394 | 0.004754 |
| *Dielma* | 0 | 0.001557 | 0 | 0 | 0 | 0 | 0 |
| *Dorea* | 0.085899 | 0.092078 | 0.30494 | 0.189115 | 0.109682 | 0.129507 | 0.10646 |
| *Dysgonomonas* | 0 | 0.003943 | 0 | 0 | 0 | 0 | 0 |
| *Eggerthella* | 0 | 0.006089 | 0 | 0 | 0 | 0 | 0 |
| *Ehrlichia* | 0 | 0.000152 | 0 | 0 | 0 | 0 | 0 |
| *Eisenbergiella* | 0 | 0.032842 | 0 | 0 | 0 | 0 | 0 |
| *Elusimicrobium* | 0.000631 | 0.002234 | 0 | 0.00524 | 0 | 0 | 0 |
| *Enhydrobacter* | 0.000631 | 0 | 0 | 0 | 0 | 0 | 0 |
| *Enorma* | 0 | 0.000152 | 0 | 0 | 0 | 0 | 0 |
| *Enterobacter* | 0 | 0.006052 | 0 | 0.001072 | 0 | 0.000579 | 0 |
| *Enterococcus* | 0.006785 | 0.00617 | 0.000659 | 0.006754 | 0 | 0.008681 | 0.004167 |
| *Enterorhabdus* | 0 | 0.000682 | 0 | 0 | 0 | 0 | 0 |
| *Epulopiscium* | 0 | 0 | 0 | 0 | 0 | 0.001022 | 0.000535 |
| *Erysipelatoclostridium* | 0 | 0.003852 | 0 | 0 | 0 | 0 | 0 |
| *Erysipelothrix* | 0 | 0.008762 | 0 | 0 | 0 | 0 | 0 |
| *Escherichia* | 0 | 0.031041 | 0 | 0 | 0 | 0 | 0 |
| *Ethanoligenens* | 0 | 0.025659 | 0 | 0 | 0 | 0 | 0 |
| *Eubacterium* | 0 | 4.095257 | 0 | 0 | 0 | 0.000468 | 0.000875 |
| *Euzebya* | 0.006556 | 0 | 0 | 0 | 0 | 0 | 0 |
| *Facklamia* | 0.004615 | 0.001999 | 0.000827 | 0.08732 | 0.065539 | 0.067686 | 0.02766 |
| *Faecalibacterium* | 0.015251 | 0.281414 | 0.004543 | 0.011193 | 0.005935 | 0.013094 | 0.009129 |
| *Faecalicoccus* | 0 | 0.000165 | 0 | 0 | 0 | 0 | 0 |
| *Faecalitalea* | 0 | 0.000489 | 0 | 0 | 0 | 0 | 0 |
| *Fastidiosipila* | 0 | 0.00032 | 0 | 0 | 0 | 0 | 0 |
| *Fibrobacter* | 0.28999 | 0.002337 | 0 | 0 | 0 | 0 | 0 |
| *Filifactor* | 0 | 0 | 0 | 0 | 0 | 0.000468 | 0 |
| *Fimbriimonas* | 0.000656 | 0 | 0 | 0 | 0 | 0 | 0 |
| *Finegoldia* | 0 | 0.000152 | 0 | 0 | 0 | 0 | 0 |
| *Flavisolibacter* | 0.005245 | 0 | 0 | 0 | 0 | 0 | 0 |
| *Flavobacterium* | 0 | 0.056277 | 0 | 0 | 0 | 0 | 0 |
| *Flavonifractor* | 0 | 0.091811 | 0 | 0 | 0 | 0 | 0 |
| *Flexibacter* | 0 | 0.000196 | 0 | 0 | 0 | 0 | 0 |
| *Flexispira* | 0.440854 | 0.000316 | 0.010066 | 0.573055 | 2.661747 | 1.537857 | 3.245373 |
| *Fluviicola* | 0 | 2.735234 | 0 | 0 | 0 | 0 | 0 |
| *Fusibacter* | 0 | 0 | 0 | 0.000443 | 0 | 0 | 0.000468 |
| *Fusicatenibacter* | 0 | 0.060715 | 0 | 0 | 0 | 0 | 0 |
| *Fusobacterium* | 0.002171 | 0.000641 | 0 | 0.001005 | 0.007304 | 0.017543 | 0.000937 |
| *Galbibacter* | 0 | 0.000152 | 0 | 0 | 0 | 0 | 0 |
| *Gemella* | 0 | 0.00124 | 0 | 0 | 0 | 0 | 0 |
| *Gemmata* | 0.001967 | 0 | 0.001655 | 0 | 0 | 0 | 0 |
| *Gemmatimonas* | 0.001967 | 0 | 0 | 0 | 0 | 0 | 0 |
| *Geobacter* | 0 | 0.000819 | 0 | 0 | 0 | 0 | 0 |
| *Geodermatophilus* | 0.001311 | 0 | 0 | 0 | 0 | 0 | 0 |
| *Geopsychrobacter* | 0 | 0.000156 | 0 | 0 | 0 | 0 | 0 |
| *Globicatella* | 0 | 0.000321 | 0 | 0 | 0 | 0 | 0 |
| *Gloeobacter* | 0 | 0.021241 | 0 | 0 | 0 | 0 | 0 |
| *Gordonibacter* | 0 | 0.003168 | 0 | 0 | 0 | 0 | 0 |
| *Gracilibacter* | 0 | 0.008368 | 0 | 0 | 0 | 0 | 0 |
| *Gramella* | 0 | 0 | 0 | 0 | 0 | 0.000468 | 0.001749 |
| *Granulicatella* | 0.003174 | 0.002484 | 0.001402 | 0.00414 | 0.000478 | 0.003141 | 0.003292 |
| *Haemophilus* | 0 | 0.000506 | 0 | 0.001064 | 0.001267 | 0 | 0 |
| *Halichoeres* | 0 | 0.023949 | 0 | 0 | 0 | 0 | 0 |
| *Hallella* | 0 | 0.000156 | 0 | 0 | 0 | 0 | 0 |
| *Halomonas* | 0 | 0 | 0 | 0.000443 | 0 | 0 | 0 |
| *Haloplasma* | 0 | 0.000196 | 0 | 0 | 0 | 0 | 0 |
| *Helicobacter* | 0.63286 | 2.948633 | 0.909234 | 1.199171 | 2.354781 | 2.227972 | 1.87042 |
| *Hespellia* | 0 | 0.005959 | 0 | 0 | 0 | 0 | 0 |
| *Hirschia* | 0 | 0.000714 | 0 | 0 | 0 | 0 | 0 |
| *Holdemanella* | 0 | 0.006905 | 0 | 0 | 0 | 0 | 0 |
| *Holdemania* | 0 | 0.004232 | 0 | 0 | 0.001913 | 0 | 0.003011 |
| *Howardella* | 0 | 0.000522 | 0 | 0 | 0 | 0 | 0 |
| *Hydrogenoanaerobacterium* | 0 | 0.010388 | 0 | 0 | 0 | 0 | 0 |
| *Hymenobacter* | 0 | 0.000472 | 0 | 0 | 0 | 0 | 0 |
| *Hyphomicrobium* | 0.001311 | 0 | 0 | 0 | 0 | 0 | 0 |
| *Hypotrichomonas* | 0 | 0.06218 | 0 | 0 | 0 | 0 | 0 |
| *Iamia* | 0.000656 | 0 | 0 | 0 | 0 | 0 | 0 |
| *Intestinibacter* | 0 | 0.000325 | 0 | 0 | 0 | 0 | 0 |
| *Intestinimonas* | 0 | 0.454961 | 0 | 0 | 0 | 0 | 0 |
| *Jeotgalibacillus* | 0 | 0.001693 | 0 | 0 | 0 | 0 | 0 |
| *Jeotgalicoccus* | 0.144742 | 0.00807 | 0.110158 | 0.051358 | 0.012236 | 0.048472 | 0.018717 |
| *Johnsonella* | 0 | 0.005693 | 0 | 0 | 0 | 0 | 0 |
| *Kaistibacter* | 0.000656 | 0 | 0 | 0 | 0 | 0 | 0 |
| *Kaistobacter* | 0.160622 | 0 | 0.000828 | 0 | 0.00037 | 0.000579 | 0 |
| *Klebsiella* | 0 | 0.003393 | 0.000659 | 0 | 0 | 0.001515 | 0 |
| *Kopriimonas* | 0 | 0.031419 | 0 | 0 | 0 | 0 | 0 |
| *Kribbella* | 0.001311 | 0 | 0 | 0 | 0 | 0 | 0 |
| KSA1 | 0.000631 | 0 | 0 | 0 | 0 | 0 | 0 |
| *Lachnoanaerobaculum* | 0 | 0.008493 | 0 | 0 | 0 | 0 | 0 |
| *Lachnobacterium* | 0.037508 | 0.002181 | 0.002313 | 0.028536 | 0.067296 | 0.068474 | 0.01183 |
| *Lachnoclostridium* | 0 | 9.827545 | 0 | 0 | 0 | 0 | 0 |
| *Lachnospira* | 0.000677 | 0.046132 | 0.026137 | 0.005945 | 0.013724 | 0.00442 | 0.000535 |
| *Lactobacillus* | 0.387907 | 2.411202 | 1.248502 | 0.907549 | 0.342007 | 1.285398 | 1.043458 |
| *Lactococcus* | 0.022578 | 0.55393 | 0 | 0.013575 | 0.001185 | 0.032556 | 0.013586 |
| *Lautropia* | 0 | 0.000818 | 0 | 0 | 0 | 0 | 0 |
| *Lawsonia* | 0 | 0.000481 | 0 | 0 | 0 | 0 | 0 |
| *Lentibacillus* | 0 | 0 | 0 | 0 | 0 | 0.000468 | 0 |
| *Lentzea* | 0.0059 | 0 | 0 | 0 | 0 | 0 | 0 |
| *Leuconostoc* | 0.000677 | 0.000308 | 0 | 0.001872 | 0.00037 | 0.002894 | 0.002674 |
| *Listeria* | 0 | 0.001412 | 0 | 0 | 0 | 0 | 0 |
| *Litorilinea* | 0 | 0 | 0 | 0 | 0 | 0.000579 | 0 |
| *Luteimonas* | 0 | 0.000942 | 0.010252 | 0 | 0.014243 | 0.001012 | 0 |
| *Luteolibacter* | 0.000656 | 0 | 0 | 0 | 0 | 0 | 0 |
| *Lutispora* | 0 | 0.002971 | 0 | 0 | 0 | 0 | 0 |
| *Lysinibacillus* | 0 | 0 | 0 | 0.003741 | 0 | 0.001158 | 0.000535 |
| *Lysobacter* | 0.019012 | 0.003483 | 0.000742 | 0 | 0 | 0.000884 | 0 |
| *Mangroviflexus* | 0 | 0.056328 | 0 | 0 | 0 | 0 | 0 |
| *Marinilabilia* | 0 | 0.000193 | 0 | 0 | 0 | 0 | 0 |
| *Marinobacter* | 0 | 0 | 0 | 0 | 0 | 0.000468 | 0 |
| *Marinomonas* | 0.000631 | 0 | 0 | 0.000443 | 0.00037 | 0 | 0.001749 |
| *Marivirga* | 0 | 0.000903 | 0 | 0 | 0 | 0 | 0 |
| *Marvinbryantia* | 0 | 0.211435 | 0 | 0 | 0 | 0 | 0 |
| *Megamonas* | 0 | 0.092892 | 0.000827 | 0.000443 | 0 | 0.000468 | 0 |
| *Megasphaera* | 0.014798 | 0.008694 | 0.002483 | 0.021862 | 0.006833 | 0.019486 | 0.013308 |
| *Meniscus* | 0 | 0.001794 | 0 | 0 | 0 | 0 | 0 |
| *Mesorhizobium* | 0.000656 | 0 | 0 | 0 | 0 | 0 | 0 |
| *Methanobrevibacter* | 0.000631 | 0.001119 | 0.086129 | 0.002324 | 0 | 0 | 0.001074 |
| *Methylibium* | 0.000656 | 0 | 0 | 0.000357 | 0 | 0 | 0 |
| *Methylocella* | 0 | 0.00016 | 0 | 0 | 0 | 0 | 0 |
| *Methylonatrum* | 0 | 0.000193 | 0 | 0 | 0 | 0 | 0 |
| *Microbacterium* | 0 | 0 | 0 | 0.000531 | 0 | 0 | 0 |
| *Microlunatus* | 0.000656 | 0 | 0 | 0 | 0 | 0 | 0 |
| *Mitsuokella* | 0.002526 | 0.007707 | 0 | 0.005314 | 0.000311 | 0.002809 | 0.004167 |
| *Modestobacter* | 0.001967 | 0 | 0 | 0 | 0 | 0 | 0 |
| *Mogibacterium* | 0 | 0.000304 | 0 | 0.00126 | 0 | 0.001626 | 0.001405 |
| *Morganella* | 0 | 0.000813 | 0 | 0 | 0 | 0 | 0 |
| *Moryella* | 0 | 0.072556 | 0 | 0.001063 | 0 | 0 | 0 |
| *Mucilaginibacter* | 0 | 0.00535 | 0 | 0 | 0 | 0 | 0 |
| *Mucispirillum* | 0.08111 | 0.024929 | 0.090076 | 0.294028 | 0.218214 | 0.16397 | 0.377722 |
| *Muricola* | 0 | 0 | 0 | 0 | 0 | 0 | 0.000875 |
| *Mycobacterium* | 0.001967 | 0 | 0 | 0 | 0 | 0 | 0 |
| *Mycoplana* | 0.004589 | 0 | 0 | 0.001063 | 0 | 0 | 0 |
| *Mycoplasma* | 0.018668 | 0.007705 | 0.009652 | 0.009901 | 0 | 0 | 0.102479 |
| *Natranaerovirga* | 0 | 0.01964 | 0 | 0 | 0 | 0 | 0 |
| *Necropsobacter* | 0.004901 | 0.000193 | 0.00561 | 0.004031 | 0.000741 | 0 | 0.001071 |
| *Nitrospira* | 0.003934 | 0 | 0 | 0.000443 | 0 | 0 | 0 |
| *Nocardioides* | 0.001967 | 0 | 0 | 0 | 0 | 0 | 0 |
| *Nosocomiicoccus* | 0 | 0.000477 | 0 | 0 | 0 | 0 | 0 |
| *Novispirillum* | 0 | 0.000361 | 0 | 0 | 0 | 0 | 0 |
| *Novosphingobium* | 0.003934 | 0 | 0 | 0 | 0.000681 | 0 | 0 |
| *Oceanospirillum* | 0.002526 | 0 | 0 | 0.0031 | 0.000407 | 0.001515 | 0.005784 |
| *Ochrobactrum* | 0 | 0 | 0 | 0 | 0 | 0.000579 | 0 |
| *Odoribacter* | 1.09185 | 1.797592 | 0.49174 | 0.634297 | 0.163742 | 0.27322 | 0.48479 |
| *Oligella* | 0.031089 | 0.002216 | 0.107937 | 1.329016 | 1.260812 | 1.969084 | 0.31219 |
| *Oligosphaera* | 0 | 0.000349 | 0 | 0 | 0 | 0 | 0 |
| *Olivibacter* | 0 | 0.000681 | 0 | 0 | 0 | 0 | 0 |
| *Olsenella* | 0 | 0.000477 | 0 | 0 | 0 | 0 | 0 |
| *Opitutus* | 0 | 0.000321 | 0 | 0 | 0 | 0 | 0 |
| *Oribacterium* | 0 | 0.000329 | 0 | 0 | 0 | 0 | 0 |
| *Oscillibacter* | 0 | 0.025415 | 0 | 0 | 0 | 0 | 0 |
| *Oscillospira* | 7.188583 | 2.36369 | 3.178062 | 3.862039 | 5.561893 | 7.212384 | 4.778661 |
| *Owenweeksia* | 0 | 0.000559 | 0 | 0 | 0 | 0 | 0 |
| *Oxobacter* | 0 | 0.000637 | 0 | 0 | 0 | 0 | 0 |
| p-75-a5 | 0.001894 | 0 | 0 | 0.001685 | 0.001481 | 0.002893 | 0.004282 |
| *Paenibacillus* | 0.000656 | 0.000156 | 0 | 0.000531 | 0 | 0.000941 | 0 |
| *Paludibacter* | 0 | 0.229044 | 0 | 0 | 0 | 0 | 0 |
| *Papillibacter* | 0 | 0.062966 | 0 | 0 | 0 | 0 | 0 |
| *Parabacteroides* | 1.40673 | 0.27918 | 0.355338 | 1.612288 | 1.629801 | 1.482441 | 1.616121 |
| *Paracoccus* | 0 | 0 | 0 | 0 | 0 | 0.001012 | 0.001606 |
| *Paraeggerthella* | 0 | 0.000156 | 0 | 0 | 0 | 0 | 0 |
| *Parapedobacter* | 0 | 0.000502 | 0 | 0 | 0 | 0 | 0 |
| *Paraprevotella* | 0.291521 | 0.050591 | 0.001402 | 0.74817 | 0.521511 | 0.740798 | 0.540604 |
| *Parasporobacterium* | 0 | 0.000857 | 0 | 0 | 0 | 0 | 0 |
| *Parasutterella* | 0 | 0.033484 | 0 | 0 | 0 | 0 | 0 |
| *Parvibacter* | 0 | 0.000316 | 0 | 0 | 0 | 0 | 0 |
| *Parvibaculum* | 0 | 0.076977 | 0 | 0 | 0 | 0 | 0 |
| *Parvularcula* | 0 | 0.001315 | 0 | 0 | 0 | 0 | 0 |
| *Pasteurella* | 0 | 0.004482 | 0 | 0 | 0 | 0 | 0 |
| *Pediococcus* | 0 | 0.000308 | 0 | 0 | 0 | 0 | 0 |
| *Pedobacter* | 0.000656 | 0.005049 | 0 | 0 | 0 | 0 | 0 |
| *Pedomicrobium* | 0.002622 | 0 | 0 | 0 | 0 | 0 | 0 |
| *Pentatrichomonas* | 0 | 0.00554 | 0 | 0 | 0 | 0 | 0 |
| *Peptoclostridium* | 0 | 0.004808 | 0 | 0 | 0 | 0 | 0 |
| *Peptococcus* | 0 | 0.015484 | 0 | 0 | 0 | 0 | 0 |
| *Phascolarctobacterium* | 0.385538 | 0.003842 | 0.001402 | 0.202218 | 0.538814 | 0.009719 | 0.257197 |
| *Pilimelia* | 0.000631 | 0 | 0 | 0 | 0 | 0 | 0 |
| *Pirellula* | 0.003934 | 0 | 0 | 0 | 0 | 0 | 0 |
| *Planctomyces* | 0.001311 | 0.001007 | 0 | 0 | 0 | 0 | 0 |
| *Pleomorphomonas* | 0 | 0 | 0 | 0 | 0.000957 | 0 | 0 |
| *Pontibacter* | 0 | 0.017791 | 0 | 0 | 0 | 0 | 0 |
| *Porphyromonas* | 0 | 0.052821 | 0 | 0 | 0 | 0 | 0.000669 |
| *Prevotella* | 20.10735 | 2.507412 | 19.01524 | 25.7094 | 16.24774 | 14.83699 | 25.44191 |
| *Prolixibacter* | 0 | 0.001666 | 0 | 0 | 0 | 0 | 0 |
| *Propionibacterium* | 0 | 0 | 0 | 0.000384 | 0 | 0.000468 | 0.000537 |
| *Proteiniphilum* | 0 | 0.00164 | 0 | 0 | 0 | 0 | 0 |
| *Proteus* | 0 | 0.019063 | 0 | 0 | 0 | 0 | 0 |
| *Pseudidiomarina* | 0 | 0 | 0 | 0 | 0 | 0 | 0.000875 |
| *Pseudoalteromonas* | 0.001263 | 0 | 0 | 0.001771 | 0.00037 | 0.00515 | 0.001749 |
| *Pseudobutyrivibrio* | 0 | 0.039774 | 0 | 0 | 0 | 0 | 0 |
| *Pseudoflavonifractor* | 0 | 1.122277 | 0 | 0 | 0 | 0 | 0 |
| *Pseudomonas* | 0.004061 | 0 | 0.001318 | 0.002776 | 0.004179 | 0.003915 | 0.004155 |
| *Pseudonocardia* | 0.001311 | 0 | 0 | 0 | 0 | 0 | 0 |
| *Psychrobacter* | 0.025901 | 0 | 0.039286 | 0.047464 | 0.072399 | 0.059138 | 0.00642 |
| *Ramlibacter* | 0.001311 | 0 | 0 | 0 | 0 | 0 | 0 |
| *Raphanus* | 0 | 0 | 0 | 0 | 0.000478 | 0 | 0 |
| rc4-4 | 0.029598 | 0 | 0.020284 | 0.153594 | 0.319642 | 0.169705 | 0.102726 |
| RFN20 | 0.005324 | 0 | 0 | 0.036635 | 0 | 0.004941 | 0.036309 |
| *Rhizobium* | 0 | 0.000156 | 0 | 0 | 0 | 0.000941 | 0 |
| *Rhodobacter* | 0 | 0.001334 | 0 | 0 | 0 | 0 | 0 |
| *Rhodocista* | 0 | 0.001856 | 0 | 0 | 0 | 0 | 0 |
| *Rhodococcus* | 0.001311 | 0.000995 | 0.000827 | 0.001072 | 0 | 0 | 0 |
| *Rhodocyclus* | 0 | 0.000165 | 0 | 0 | 0 | 0 | 0 |
| *Rhodoplanes* | 0.013768 | 0 | 0 | 0 | 0 | 0.000941 | 0.000669 |
| *Rikenella* | 0 | 1.630705 | 0 | 0 | 0 | 0 | 0 |
| *Robinsoniella* | 0 | 0.534411 | 0 | 0 | 0 | 0 | 0 |
| *Roseburia* | 0.120867 | 2.638773 | 0.092513 | 0.04459 | 0.031379 | 0.043452 | 0.057244 |
| *Rothia* | 0 | 0.000517 | 0.000828 | 0 | 0 | 0.000468 | 0 |
| *Rubrobacter* | 0.007212 | 0.002213 | 0 | 0.000357 | 0.000478 | 0.001591 | 0 |
| *Ruminiclostridium* | 0 | 0.547529 | 0 | 0 | 0 | 0 | 0 |
| *Ruminobacillus* | 0 | 0.000624 | 0 | 0 | 0 | 0 | 0 |
| *Ruminococcus* | 0.782984 | 2.495224 | 1.117376 | 0.524176 | 0.557634 | 1.121251 | 0.80684 |
| *Saccharibacter* | 0 | 0.000589 | 0 | 0 | 0 | 0 | 0 |
| *Saccharofermentans* | 0 | 0.005047 | 0 | 0 | 0 | 0 | 0 |
| *Saccharopolyspora* | 0 | 0 | 0.000828 | 0 | 0 | 0 | 0 |
| *Salinimicrobium* | 0 | 0 | 0 | 0.000443 | 0 | 0 | 0 |
| *Schwartzia* | 0 | 1.659672 | 0 | 0 | 0 | 0 | 0 |
| *Selenomonas* | 0 | 0.160038 | 0 | 0 | 0 | 0 | 0 |
| *Senegalimassilia* | 0 | 0.000357 | 0 | 0 | 0 | 0 | 0 |
| *Serratia* | 0.000677 | 0 | 0 | 0 | 0.000741 | 0 | 0.001674 |
| *Shewanella* | 0 | 0 | 0 | 0 | 0.000425 | 0 | 0 |
| *Shigella* | 0 | 0.00241 | 0 | 0 | 0 | 0 | 0 |
| *Skermanella* | 0.000656 | 0 | 0 | 0 | 0 | 0 | 0 |
| *Slackia* | 0 | 0.003619 | 0 | 0 | 0 | 0 | 0 |
| SMB53 | 1.369814 | 0 | 4.964779 | 1.841635 | 2.146036 | 1.994122 | 2.286822 |
| *Solitalea* | 0 | 0.009015 | 0 | 0 | 0 | 0 | 0 |
| *Solobacterium* | 0 | 0.00016 | 0 | 0 | 0 | 0 | 0 |
| *Sphingobacterium* | 0.001598 | 0.214474 | 0 | 0.002657 | 0.001913 | 0.003536 | 0 |
| *Sphingomonas* | 0.026245 | 0.00016 | 0 | 0.000357 | 0.000621 | 0.000579 | 0 |
| *Spirochaeta* | 0 | 0.000304 | 0 | 0 | 0 | 0 | 0 |
| *Spiroplasma* | 0 | 0.000991 | 0 | 0 | 0 | 0 | 0 |
| *Sporanaerobacter* | 0 | 0.009268 | 0 | 0 | 0 | 0 | 0 |
| *Sporobacter* | 0 | 0.143344 | 0 | 0 | 0 | 0 | 0 |
| *Sporolactobacillus* | 0 | 0.000156 | 0 | 0 | 0 | 0 | 0 |
| *Sporosarcina* | 0 | 0 | 0 | 0 | 0.00037 | 0 | 0.000468 |
| *Stanieria* | 0 | 0 | 0 | 0 | 0.000478 | 0 | 0 |
| *Staphylococcus* | 0.021475 | 0.022371 | 0.099159 | 0.039324 | 0.020816 | 0.017773 | 0.034732 |
| *Steroidobacter* | 0.003278 | 0 | 0 | 0 | 0 | 0 | 0 |
| *Stomatobaculum* | 0 | 0.005679 | 0 | 0 | 0 | 0 | 0 |
| *Streptococcus* | 0.052508 | 0.047035 | 0.058735 | 0.065761 | 0.029031 | 0.061948 | 0.043559 |
| *Streptomyces* | 0.001311 | 0 | 0 | 0 | 0 | 0 | 0 |
| *Subdoligranulum* | 0 | 0.112195 | 0 | 0 | 0 | 0 | 0 |
| *Succinivibrio* | 0 | 0.012551 | 0 | 0.0008 | 0.000956 | 0 | 0 |
| *Sutterella* | 0.049918 | 0.010061 | 0.018805 | 0.428367 | 0.393855 | 0.444289 | 0.254797 |
| *Symbiobacterium* | 0 | 0.000328 | 0 | 0 | 0 | 0 | 0 |
| *Synergistes* | 0 | 0.000661 | 0 | 0 | 0 | 0 | 0 |
| *Syntrophococcus* | 0 | 1.111271 | 0 | 0 | 0 | 0 | 0 |
| *Tannerella* | 0 | 2.350988 | 0 | 0 | 0 | 0 | 0 |
| *Tetratrichomonas* | 0 | 0.001472 | 0 | 0 | 0 | 0 | 0 |
| *Thermosynechococcus* | 0 | 0 | 0 | 0 | 0 | 0 | 0.000669 |
| *Thermosyntropha* | 0 | 0.000156 | 0 | 0 | 0 | 0 | 0 |
| *Thiothrix* | 0 | 0.00175 | 0 | 0 | 0 | 0 | 0 |
| *Treponema* | 0.28358 | 11.41527 | 1.064623 | 4.356182 | 0.09725 | 0.437542 | 0.988296 |
| *Trichomitopsis* | 0 | 0.000337 | 0 | 0 | 0 | 0 | 0 |
| *Tritrichomonas* | 0 | 0.026266 | 0 | 0 | 0 | 0 | 0 |
| *Turicibacter* | 1.474283 | 1.427008 | 1.781808 | 0.787655 | 0.114189 | 0.054699 | 0.475897 |
| *Tyzzerella* | 0 | 0.114935 | 0 | 0 | 0 | 0 | 0 |
| *Unclassified* | 48.31345 | 0 | 47.99401 | 37.74164 | 43.88638 | 45.95383 | 42.40846 |
| vadinCA11 | 0.000617 | 0 | 0 | 0.001005 | 0.00116 | 0 | 0.002823 |
| *Vagococcus* | 0 | 0.000196 | 0 | 0.000357 | 0 | 0 | 0 |
| *Veillonella* | 0 | 0.008754 | 0.007671 | 0.001948 | 0.007514 | 0 | 0.001412 |
| *Vibrio* | 0 | 0.001027 | 0 | 0 | 0 | 0 | 0 |
| *Victivallis* | 0 | 0.000354 | 0 | 0 | 0 | 0 | 0 |
| *Virgisporangium* | 0.000656 | 0 | 0 | 0 | 0 | 0 | 0 |
| *Weissella* | 0 | 0.025141 | 0 | 0 | 0 | 0 | 0 |
| *Wolinella* | 0 | 0 | 0 | 0 | 0 | 0 | 0.001606 |
| *Yaniella* | 0.005181 | 0.00197 | 0.094647 | 0.014096 | 0.003742 | 0.006809 | 0.00637 |
| YRC22 | 0.896473 | 0 | 4.474435 | 1.227931 | 0.855884 | 0.691709 | 0.883839 |
| 5-7N15 | 0 | 0 | 0.000742 | 0.000443 | 0.001219 | 0 | 0.000537 |
| A17 | 0.002622 | 0 | 0 | 0 | 0 | 0 | 0 |

NCD, normal chow diet; NCD-T, normal chow diet + atorvastatin treatment; HFD, high-fat diet; 5 mg/kg, HFD + 5mg/kg atorvastatin; 10 mg/kg, HFD + 10 mg/kg atorvastatin; 15 mg/kg, HFD + 15 mg/kg atorvastatin; 20 mg/kg, HFD + 20 mg/kg atorvastatin.

**Supplementary Table 4: List of the families that were shared and unique between NCD and HFD group.**

| **Groups** | **Total** | **Families** |
| --- | --- | --- |
| **HFD NCD** | 56 | Gemmataceae, Brachyspiraceae, Veillonellaceae, Moraxellaceae, Pseudomonadaceae, Porphyromonadaceae, Bacillaceae, Desulfovibrionaceae, Bacteroidaceae, Lactobacillaceae, Streptococcaceae, Corynebacteriaceae, Enterobacteriaceae, Deferribacteraceae, Bifidobacteriaceae, Alcaligenaceae, Staphylococcaceae, C111, Peptococcaceae, Aerococcaceae, F16, Anaeroplasmataceae, S24-7, Peptostreptococcaceae, Lachnospiraceae, Coriobacteriaceae, Enterococcaceae, Pasteurellaceae, Prevotellaceae, Elusimicrobiaceae, Carnobacteriaceae, Helicobacteraceae, Nocardiaceae, Clostridiaceae, Pseudonocardiaceae, Micrococcaceae, Barnesiellaceae, Rikenellaceae, Sphingomonadaceae, Mycoplasmataceae, Christensenellaceae, Yaniellaceae, Methanobacteriaceae, Odoribacteraceae, Erysipelotrichaceae, Verrucomicrobiaceae, Turicibacteraceae, Ruminococcaceae, Spirochaetaceae, Dehalobacteriaceae, Paraprevotellaceae, Xanthomonadaceae, Rhizobiaceae, Campylobacteraceae, Mogibacteriaceae, Victivallaceae |
| **HFD** | 3 | Microbacteriaceae, Planococcaceae, Gemellaceae |
| **NCD** | 76 | NB1-i, Pseudoalteromonadaceae, R4-45B, RF16, Fibrobacteraceae, Chthoniobacteraceae, Myxococcaceae, PRR-10, Nocardioidaceae, Paenibacillaceae, Cryomorphaceae, Kineosporiaceae, Bradyrhizobiaceae, Hyphomonadaceae, Erythrobacteraceae, Solibacteraceae, Gemmatimonadaceae, MVS-65, Cytophagaceae, mb2424, Streptomycetaceae, Propionibacteriaceae, RB40, Syntrophobacteraceae, Comamonadaceae, Desulfobacteraceae, Neisseriaceae, Chitinophagaceae, Mycobacteriaceae, Frankiaceae, Sinobacteraceae, Planctomycetaceae, Methanomassiliicoccaceae, RFP12, Gaiellaceae, Leuconostocaceae, Euzebyaceae, Balneolaceae, Micromonosporaceae, Saprospiraceae, Chromatiaceae, Rhodobacteraceae, Sporichthyaceae, Caulobacteraceae, Cystobacteraceae, 0319-6A21, Phyllobacteriaceae, Rubrobacteraceae, Cellulomonadaceae, Ellin6075, Nitrospiraceae, Nitrosomonadaceae, Koribacteraceae, Eubacteriaceae, Rhodobiaceae, Pirellulaceae, Thiotrichaceae, OM27, Geodermatophilaceae, Rhodospirillaceae, Hyphomicrobiaceae, Solirubrobacteraceae, Kouleothrixaceae, Fimbriimonadaceae, Beijerinckiaceae, Weeksellaceae, Chlamydiaceae, A4b, Oceanospirillaceae, EB1017, Fusobacteriaceae, Patulibacteraceae, Iamiaceae, Oxalobacteraceae, Actinosynnemataceae, Sphingobacteriaceae |

Abbreviations: NCD, normal chow diet; HFD, high-fat diet.
